# Transparent Quasi-Random Structures for Multimodal Light Trapping in Ultrathin Solar Cells with Broad Engineering Tolerance

**DOI:** 10.1021/acsphotonics.2c00472

**Published:** 2022-06-23

**Authors:** Eduardo Camarillo Abad, Hannah J. Joyce, Louise C. Hirst

**Affiliations:** † Department of Physics, University of Cambridge, Cambridge, CB3 0HE, United Kingdom; ‡ Department of Engineering, University of Cambridge, Cambridge, CB3 0FA, United Kingdom; § Department of Materials Science and Metallurgy, University of Cambridge, Cambridge, CB3 0FS, United Kingdom

**Keywords:** light harvesting, ultrathin photovoltaics, correlated disorder, photonic crystal, GaAs

## Abstract

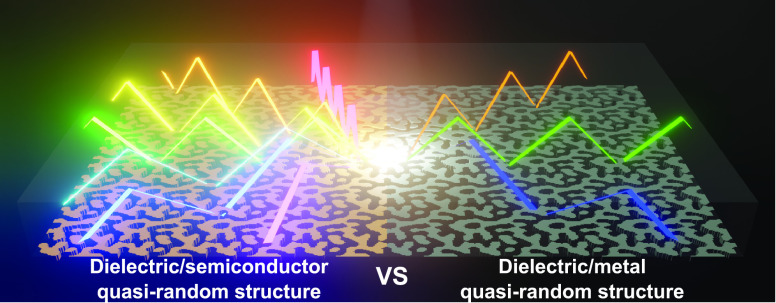

Waveguide modes are well-known to be a valuable light-trapping resource for absorption enhancement in solar cells. However, their scarcity in the thinnest device stacks compromises the multiresonant performance required to reach the highest efficiencies in ultrathin devices. We demonstrate that enriching the modal structure on such reduced length-scales is possible by integrating transparent semiconductor/dielectric scattering structures to the device architecture as opposed to more widely studied metallic textures. This phenomenon allows transparent quasi-random structures to emerge as strong light-trapping candidates for ultrathin solar cells, given that their broad scattering profiles are well-suited to exploit the increased number of waveguide modes for multiresonant absorption enhancement. A thorough study of the design space of quasi-random textures comprising more than 1500 designs confirms the superiority of transparent structures over a metallic embodiment, identifies broad and flexible design requirements to achieve optimal performances, and demonstrates photon harvesting capabilities leading to 20% efficiency with an 80 nm GaAs absorber. Our light-trapping strategy can be applied to a wide range of material systems and device architectures, is compatible with scalable low-cost fabrication techniques, and can assist current trends to reach the highest efficiencies in ever-thinner photovoltaics.

## Introduction

Growing efforts to reduce photovoltaic device thickness to ultrathin length-scales can be found across different material systems such as Si,^1−3^ GaAs,^4−6^ or CIGS.^7^ Motivations span from flexible form factors for simplified systems integration to cost reductions and reduced material usage for enhanced sustainability.^8^ The improved carrier collection efficiency of ultrathin solar cells also makes them particularly attractive for the implementation of materials with short carrier diffusion lengths, as well as for space applications where carrier lifetimes are degraded by exposure to radiation environments.^9^

The development of ultrathin solar cells is hindered by low efficiencies originating from the poor optical absorption that is inherent to this length-scale. Boosting the absorption of incident illumination requires employing antireflection coatings and rear mirrors in tandem with light-trapping strategies capable of introducing optical resonances across the spectrum.^10,11^ Waveguide resonances are of particular relevance given their strong field localization within the absorber layer, as well as their ubiquity across solar cells with different material systems. However, efficiency improvements by waveguide modes are compromised in ultrathin solar cells in view of the fact that thinner device stacks support fewer modes. The reduced modal structure of ultrathin devices restricts the ability to pack a collection of resonances across the spectrum as needed for high performance and broadband operation in solar cells.

Although enriching the modal structure can push the efficiency of ultrathin devices, this must be concomitant with texturing strategies that allow incident light to couple to these modes efficiently. Mode coupling requires an overlap between the characteristic spatial frequencies in the scattering profile of a textured surface and those of the waveguide modes supported by the device stack. Randomly rough surfaces can enable light-trapping mechanisms,^12−14^ but their scattering profiles approach a Lambertian distribution and lead to weak resonances together with significant escape cone losses. Ordered photonic crystals have highly localized diffraction profiles and offer strong coupling to optical modes at specific wavelengths,^15−19^ with corresponding absorption enhancements that can exceed the Lambertian limit.^20^ These structures can also be tailored to control escape cone losses, and have demonstrated potential to significantly boost device performance beyond that of equivalent planar devices under optimal conditions. However, the narrow diffraction profile of photonic crystals can only introduce limited resonances in the absorption profile. Using these structures to achieve optimal performance in ultrathin solar cells requires precise nanoscale design to ensure that strong absorption peaks are introduced in the most favorable spectral regions.^21,22^

Here we present transparent dielectric/semiconductor quasi-random (QR) gratings for light trapping in ultrathin solar cells. Our texturing strategy offers 3-fold advantage: increased number of waveguide modes in the device stack, engineered diffraction profiles that exploit the enhanced modal structure for multiresonant performance, and high efficiencies that are tolerant to design variability. These benefits are driven by the rich diffraction profiles of QR structures,^23−25^ which suppress optical losses, enable broadband operation,^26−30^ and smooth the absorption profile, obviating the need to tailor the spectral position of absorption peaks for optimal performance. Exhaustive optical modeling of more than 1500 textures in ultrathin GaAs devices allows us to identify the design space and performance trends of transparent QR designs, contrast their operation with that of more widely studied metallic textures, and compare their light-trapping mechanisms with ordered photonic crystals. This comprehensive study of the design space also unifies the varied conclusions obtained by previous works on the benefits of QR textures for ultrathin solar cells.^24,27,31−34^ Ultimately, our work exposes the correct design conditions to achieve optimal light harvesting with QR structures, even on length-scales where optical modes are scarce. In an 80 nm GaAs solar cell, transparent QR structures are capable of boosting light absorption by up to 43% compared to a planar device, and hold the potential to reach up to 20% efficiency. Our light-trapping strategy and design guidelines for high performance can be applied to a wide range of material systems and device architectures, and are capable of advancing light harvesting technologies in the thinnest length-scales while being compatible with low-cost process methods.

## Transparent versus Metallic Light Trapping in Ultrathin Devices

**Figure 1:**
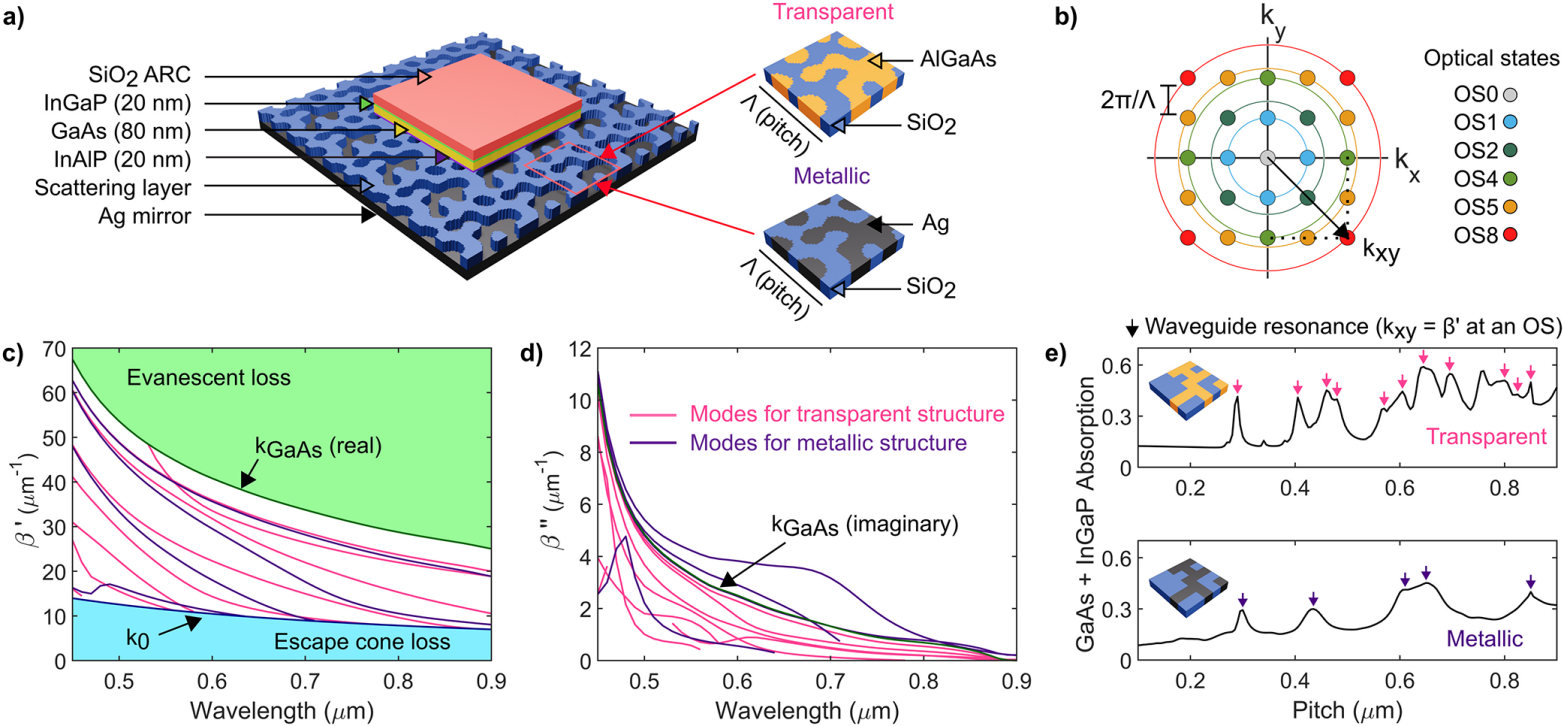
Enhanced modal structure and absorption enhancement potential of ultrathin solar cells with transparent light-trapping layers. (a) Metallic and transparent device architectures studied in this work. (b) Reciprocal space representation of the square unit cells considered for the light-trapping layer. (c) Real waveguide mode propagation constants (β′) of representative metallic (purple lines) and transparent (pink lines) devices (ARC thickness = 100 nm, grating thickness = 100 nm, fill factor = 0.5 in both cases). Transparent structures enable more waveguide modes than metallic ones. (d) Imaginary propagation constants (β″) of the waveguide modes in (c). Modes in metallic devices with higher β″ have higher losses and lower quality factors. (e) InGaP + GaAs absorption (λ = 850 nm) as a function of pitch for the representative solar cells studied in (c) and (d). Peaks correspond to coupling events to the waveguide modes in (c) at the different diffraction orders in (b).

The studied device architecture (Figure 1a) is based on the one presented in ref (21) and consists of an 80 nm GaAs active layer with 20 nm InGaP (front) and 20 nm InAlP (back) passivation layers. A SiO_2_ antireflection (ARC) coating is found on the top surface, and the scattering layer is integrated below the InAlP and on top of a back Ag mirror (ARC and grating thicknesses are not fixed). Our focus on an 80 nm device allows us to explore light trapping in the thinnest absorbers, while remaining of particular relevance for space applications owing to the radiation tolerance that has been demonstrated for GaAs solar cells on this length-scale.^9^ We study two different device concepts with either transparent (Al_0.8_Ga_0.2_As/SiO_2_) or metallic (Ag/SiO_2_) gratings with square unit cells as the scattering layer. The material combination chosen for the transparent gratings has shown to have favorable refractive index contrast for strong scattering and efficient light trapping.^35^ In particular, the low absorption coefficient of Al_0.8_Ga_0.2_As at wavelengths beyond λ ≳ 500 nm (Figure S1) allows it to be considered as a transparent material, given that the spectral region of interest for absorption enhancement in our device architecture goes from λ = 450 nm (first pass absorption is strong below this wavelength) to λ = 900 nm (GaAs absorption is negligible beyond λ ≃ 870 nm). Note that we only study the optical components of the photovoltaic device. Electrical contacts with the passivation layers would be on a different length-scale and are not considered, but localized contacting schemes with a small fraction of surface coverage have been demonstrated for these material systems.^4,14,21^

To illustrate the richer modal structure supported by an ultrathin device with a transparent texture, we use a transfer matrix method (TMM)^22^ to solve the propagation constants of the waveguide modes in representative solar cells for both transparent and metallic device concepts. The TMM considers the grating as a uniform layer with an effective index (see Methods) and decouples the modal analysis from the particular features in its unit cell. Propagation constants are of the form β = β′ + *i*β″ and correspond to the in-plane spatial frequencies of propagating waves in the modes.

Real propagation constants β′ of the waveguide modes offered by metallic and transparent textures are shown in Figure 1c. Ultrathin devices with transparent scattering layers support more waveguide modes than their metallic counterparts. This is likely due to the possibility of field propagation within the transparent scattering layer, effectively making the solar cell a thicker waveguide than a device with a metallic texture where field propagation is hindered in the metal. The waveguide modes in solar cells with metallic textures also have higher optical losses and lower quality factors (ratio of energy stored to power dissipated in the mode) as a consequence of their higher imaginary components β″ (Figure 1d).^36,37^

The modal structure of the ultrathin device stack has a strong impact on the absorption enhancement offered by a textured surface. Figure 1e shows the absorption in the ultrathin active layer of transparent and metallic device concepts as a function of the pitch of a representative grating design at λ = 850 nm (close to the GaAs band gap). Absorption is calculated for normally incident light with rigorous coupled-wave analysis (RCWA) simulations where the full device architecture is modeled and no effective indices are assumed. Varying the pitch allows incident light to access different waveguide modes supported by the device stack, given that the pitch defines the in-plane spatial frequencies *k*_*xy*_ of diffracted waves and mode coupling requires these waves to meet the phase matching condition *k*_*xy*_ = β′. As a consequence, studying the absorption as a function of pitch will show a collection of absorption peaks, each corresponding to a coupling event of a diffraction order to a waveguide mode that enhances the field in the ultrathin stack. The particular relationship between *k*_*xy*_ and the pitch for different diffraction orders is (for normal light incidence and square unit cells):
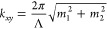
1where Λ is the pitch (periodicity) of the unit cell and *m*_1_ and *m*_2_ are pairs of integers which define diffraction orders (also referred to as optical states). Throughout this work we group diffraction orders with equivalent *k*_*xy*_ in sets that we label OSx, where x is their corresponding value inside the square root in eq 1 (see Figure 1b).

The ultrathin solar cell with a transparent grating has an increased collection of absorption peaks due to its richer modal structure (Figure 1e). These absorption peaks appear at pitches where eq 1 predicts coupling to the waveguide modes in Figure 1c as a consequence of different OS meeting the phase matching condition *k*_*xy*_ = β′ (see Supporting Information, Discussion 1 for a direct correlation). Absorption peaks in the metallic case also agree with the modal structure in Figure 1c,d, appearing at the expected pitches and being scarce and broadened owing to their lower quality factors. These observations showcase the advantages of integrating transparent scattering layers to boost photon harvesting in ultrathin device stacks by enabling more waveguide resonances.

## Implementation and Design Space of QR Structures

Supporting an increased number of waveguide modes that lead to absorption enhancement is beneficial for light trapping in any solar cell with incomplete absorption of photons, and particularly in ultrathin devices with short optical path lengths. It is expected that different transparent grating designs can offer this benefit, from ordered photonic crystals to more disordered structures (Figure S2). Quasi-random (QR) gratings are of particular interest for transparent light trapping, as they have shown promising potential to exploit rich modal structures for broadband and high absorption enhancement.^24,27^ These structures are also compatible with scalable, low-cost fabrication techniques such as polymer blend lithography.^38^ QR designs are engineered to populate a vast and tailored number of diffraction orders in order to target multiple waveguide modes and provide multiresonant performances. Consequently, they are expected to be advantageous for ultrathin solar cells when made with transparent materials, as more waveguide modes will be available for the populated diffraction orders to couple to.

To demonstrate the light-trapping potential and advantages of transparent QR scattering structures in ultrathin solar cells, we perform an exhaustive study of the design space available for these textures to identify optimal performances as well as the conditions and mechanisms that lead to such favorable operation. We do this study considering both transparent and metallic QR textures in order to make robust comparisons and highlight the benefits of implementing transparent materials.

**Figure 2:**
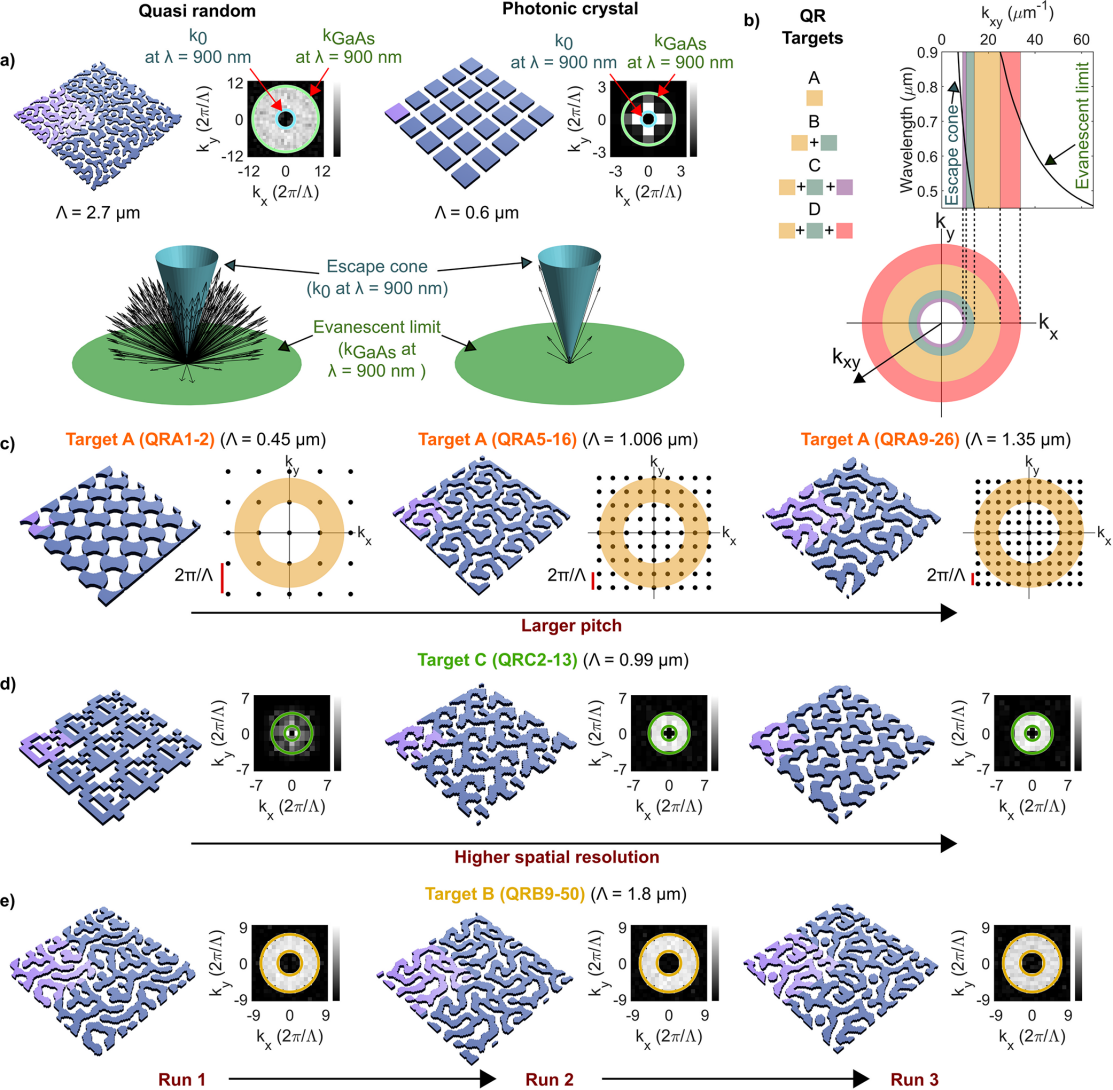
QR structure concept and design. (a) Representative QR and photonic crystal scattering structures and their Fourier spectra. The angular profile of their corresponding diffraction orders is shown for λ = 900 nm in GaAs, with the length of each arrow defined by the intensity of the corresponding diffraction order in the Fourier spectrum. (b) Target spatial frequency ranges considered for the design of QR structures. (c) For a given target, larger pitches lead to more intricate QR structures and higher densities of optical states. (d) Low resolution QR unit cells achieve poor localization of the power spectral density within the target. (e) Different real-space QR designs obtained in different runs of the design algorithm can achieve equivalent Fourier space characteristics. Scattering structures only show dielectric features and highlight those within one unit cell.

Identifying the parameter space for QR gratings begins by recognizing that the design process for these structures engineers the diffraction profile so that certain diffraction orders are preferentially occupied by diffracted light. To tailor the diffraction profile, the power spectral density of the unit cell is localized at the spatial frequencies *k*_*xy*_ of the diffraction orders whose occupation is sought (Figure 2a). This process can allow QR gratings to populate vast numbers of diffraction orders, contrary to photonic crystal gratings where the power spectral density and corresponding diffraction profile are highly localized (Figure 2a). For light-trapping purposes, the diffraction orders populated by a QR grating should be those with *k*_*xy*_ in the range from *k*_0_ (free space wavevector) to *k*_GaAs_ (wavevector in GaAs), as it is between these limits that waveguide modes and total internal reflection are available in both metallic and transparent device concepts (see Figure 1c). Additionally, it is within this range that loss mechanisms are avoided. Below *k*_0_, total internal reflection of diffracted waves does not take place and so any diffraction in that range would contribute to escape cone losses. Above *k*_GaAs_, diffracted waves are evanescent everywhere in the device and have no contributions to absorption enhancement (in the spectral range of interest for light trapping, where GaAs has the highest refractive index of all stack layers).

The wavelength dependence of waveguide modes and loss mechanisms means that the occupation of a particular diffraction order may be favorable for light trapping at some wavelengths but detrimental at others. The range of spatial frequencies covered by the diffraction orders that a QR grating populates must be carefully selected to provide beneficial light trapping across the spectral range requiring absorption enhancement. This spatial frequency range is the initial and key design parameter for QR gratings. To study this parameter and the impact of various degrees of optical losses, we initially investigate four different target spatial frequency ranges (A–D) where the diffraction profile will be localized. These targets are found in Table 1 and shown schematically in Figure 2b. Target A has no loss mechanisms within the wavelengths that require light trapping, whereas the diffraction profiles of targets B and C will lead to some escape cone losses below λ = 600 and 700 nm, respectively. Target D has escape cone losses below λ = 600 nm as well as evanescent losses above λ = 700 nm. To discuss escape cone losses, the threshold wavelength λ_th_ of a target (also included in Table 1) is defined as the wavelength below which some diffracted power lies within the escape cone. All targets apply to both metallic and transparent cases.

**Table 1:** Parameter Space Studied for Metallic and Transparent QR Unit Cell Designs

target	spatial frequency range	threshold wavelength (nm)	QR family	pitch (μm)	matrix sizes (*n* × *n*)
A	*k*_0,450 nm_–*k*_GaAs,900 nm_	450	QRA1-2	0.450	3, 13, 23, 33, 43, 53
			QRA5-16	1.006	9, 19, 29, 39, 49, 59
			QRA9-26	1.350	11, 21, 31, 41, 51, 61
B	*k*_0,600 nm_–*k*_GaAs,900 nm_	600	QRB1-5	0.600	5, 15, 25, 35, 45, 55
			QRB5-26	1.342	11, 21, 31, 41, 51, 61
			QRB9-50	1.800	15, 25, 35, 45, 55, 65
C	*k*_0,700 nm_–*k*_GaAs,900 nm_	700	QRC2-13	0.990	7, 17, 27, 37, 47, 57
			QRC5-37	1.565	13, 23, 33, 43, 53, 63
			QRC9-68	2.100	17, 27, 37, 47, 57, 67
D	*k*_0,600 nm_–*k*_GaAs,700 nm_	600	QRD1-10	0.600	7, 17, 27, 37, 47, 57
			QRD5-50	1.342	15, 25, 35, 45, 55, 65
			QRD9-90	1.800	19, 29, 39, 49, 59, 69
E	*k*_0,800 nm_–*k*_GaAs,900 nm_	800	QRE9-90	2.400	19 (metallic)
					59 (transparent)
F	*k*_0,900 nm_–*k*_GaAs,900 nm_	900	QRF9-113	2.700	21 (metallic)
					69 (transparent)

Another essential design parameter for a QR grating is the pitch of the unit cell, which determines the distribution of optical states through eq 1 (i.e., the *k*_*xy*_ values of the diffraction orders). The pitch dictates which diffraction orders are within the target under consideration and should be considered for the power spectral density localization. Larger pitches will result in higher densities of optical states, meaning more diffraction orders will lie within a target spatial frequency range (Figure 2c). To study this parameter, we select three representative pitches for each target (Table 1), all having a set of diffraction orders (OS) with *k*_*xy*_ matching the target’s lower boundary. To refer to QR gratings for different targets and with different pitches, we define QR families as QRTL-U, where T is the target under consideration, L is the OS at the target’s lower boundary, and U is the highest OS within the target (with the largest *k*_*xy*_). QR families with a higher OS at their lower boundaries have larger pitches and more intricate unit cell geometries (Figure 2c). Pitches were chosen to allow for low, intermediate, and high densities of optical states in our ultrathin devices.

Tailoring the diffraction profile of a QR family requires localizing the power spectral density of the grating at the diffraction orders within the target. This is done following a stochastic design algorithm (see Methods for full details) where the material arrangement in the unit cells is represented by *n* × *n* matrices. The algorithm aims to homogeneously localize the power spectral density among the diffraction orders within the target. Another design parameter for QR gratings is then the matrix size *n* for this design algorithm, as larger arrays increase the spatial resolution of the unit cell features. To study this parameter, for each QR family we design unit cells with six different matrix sizes, as defined in Table 1. In all cases, the lowest matrix size of a QR family corresponds to the minimum value that allows distinction between all spatial frequencies within the corresponding target. The smallest matrices have poorly localized power spectral densities due to their reduced degrees of freedom to accommodate material features (Figure 2d). On the other hand, larger matrix sizes lead to more detailed grating features and a better localization of the power spectral density among the diffraction orders within the target (see Supporting Information, Discussion 2).

Finally, two other design parameters for QR structures are the thickness of the grating and the fill factor, which we define as the fraction of the unit cell area covered with dielectric in either metallic or transparent cases. Both parameters can have an impact on diffraction efficiency and parasitic absorption. Favorable grating thicknesses were found to be constant for QR designs within a given target and so optimal values are used throughout our study (Table 2). Fill factor is initially fixed at 0.5 as this value maximizes the nonspecular power spectral density of our QR designs (see Supporting Information, Discussion 3). Variations in the fill factor will be considered after identifying light-trapping trends and favorable conditions for the design parameters defined previously.

**Table 2:** Optimal ARC and Grating Thicknesses (nm) for Different QR Targets and Grating Materials

target	ARC (metallic)	grating (metallic)	ARC (transparent)	grating (transparent)
A, B, C, E, F	80	140	100	100
D	80	120	100	120

To carry out our study of the parameter space, we design 10 different QR unit cells for each QR family and matrix size (all 720 QR designs are included in Supporting Information, Discussion 4). Studying a population of unit cells for each parameter combination is needed to obtain statistically relevant data. Being a stochastic process, the QR design algorithm can produce different structures for the same matrix size and QR family with equivalent Fourier space characteristics (Figure 2e).

The light-trapping performance of each QR unit cell design is evaluated by calculating the short circuit current density (*J*_sc_) expected for an ultrathin device (Figure 1a) with the corresponding grating. *J*_sc_ is calculated according to the following equation:
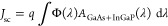
2where Φ(λ) is the photon flux in the AM1.5G solar spectrum as a function of wavelength λ, *q* is the elementary charge, and *A*_GaAs+InGaP_(λ) is the fraction of photons absorbed in the GaAs and InGaP at each wavelength, calculated with RCWA simulations (charge carriers generated in the InGaP can also be extracted as current^21^). Note that this equation assumes 100% collection of photogenerated charge carriers, a reasonable approximation given the high collection efficiency that has been experimentally demonstrated for equivalent devices.^21^ Shading and other possible losses from contacting schemes are not considered.

A flowchart depicting the different steps of our study is included in Figure S3.

## Transparent QR Structures for Robust and High Performing Ultrathin Photovoltaics

**Figure 3:**
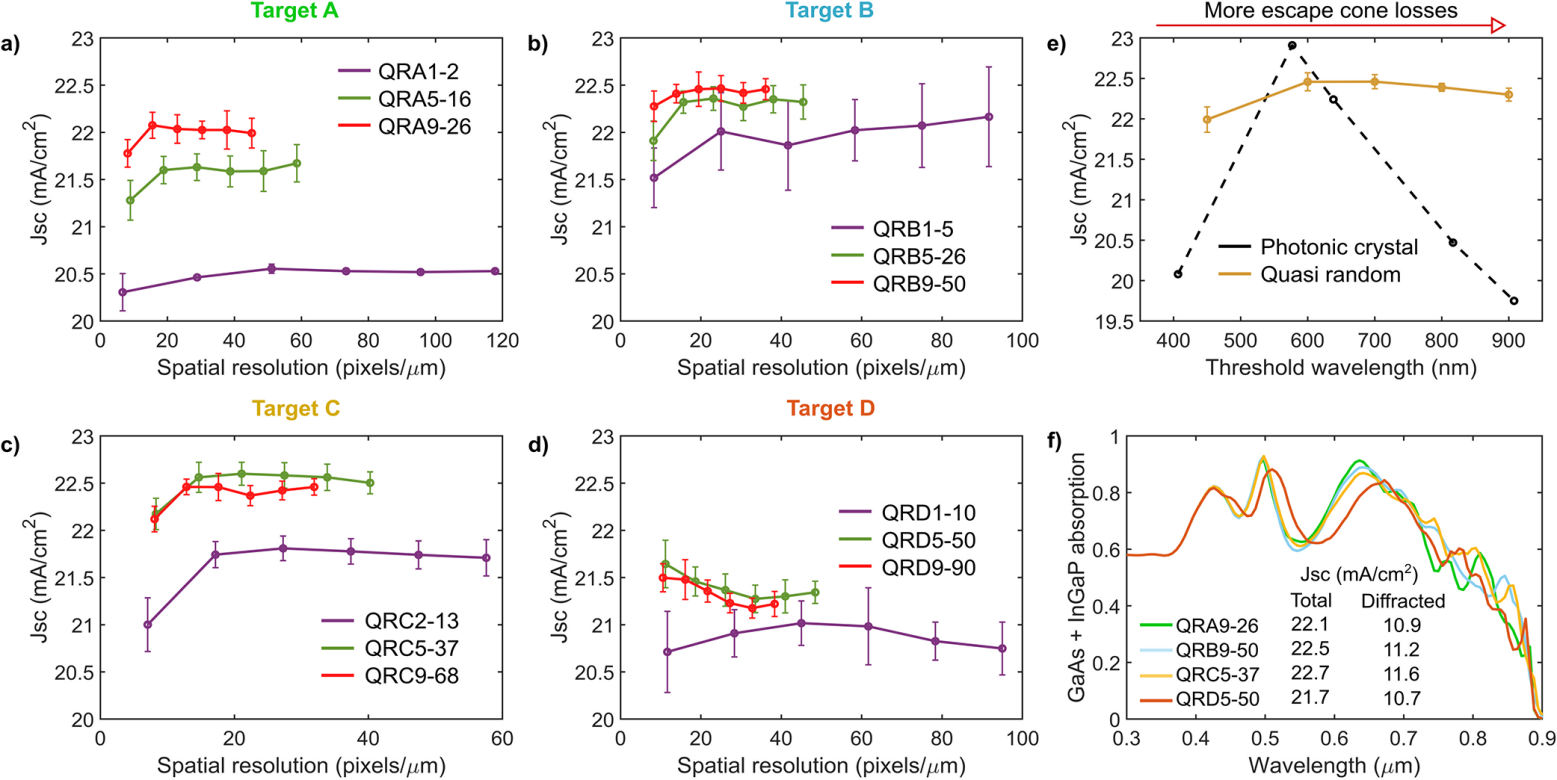
Light-trapping performance of transparent QR structures. (a–d) Average performance (*J*_sc_) of all 10 quasi-random unit cells designed for each target, QR family, and spatial resolution (matrix size), with the optimal parameters in Table 2 (error bars correspond to the standard deviation of the *J*_sc_ for a given population). (e) Performance of transparent QR structures and optimal photonic crystals as a function of the threshold wavelength, which marks the spectral limit below which power is diffracted within the escape cone. (f) Absorption (GaAs + InGaP) plots for representative devices of the best performing QR family and resolution for each target.

The performance of QR gratings for different targets, pitches (QR families) and matrix sizes (spatial resolutions) is shown in Figure 3 a-d for transparent devices. Only the average *J*_sc_ of all 10 unit cells for the same parameter combination is shown. Error bars correspond to the standard deviation of the *J*_sc_ within this population and demonstrate that, for most parameter combinations, performance remains stable for different real space designs as long as their Fourier characteristics are equivalent. Except for QRB1-5 and QRD1-10, where few optical states are found within the target region and real space designs are the least intricate (see Supporting Information, Discussion 4), in all other cases performance deviations stay below 0.3 mA/cm^2^. QRA1-2 also has few optical states within the target, but in this case, the QR design algorithm converged to the same structure for most runs and so deviations are minimal.

Performance trends show that transparent grating designs for a given QR family are tolerant to variations in the spatial resolution of the unit cell features. Except for QRD5-50 and QRD9-90, all other QR families show stable performances upon varying this parameter. Only the lowest spatial resolutions show reduced performances, given that the smallest matrices have the least optimized diffraction profiles. With increased diffraction at OS with *k*_*xy*_ outside the corresponding target (Figure 2d), the smallest arrays entail more optical losses. As for QRD5-50 and QRD9-90, their slightly reduced performances as resolution is increased might imply that increased diffraction outside target D can be beneficial. In low-resolution structures with poorly localized diffraction profiles, reducing the population of the evanescent diffraction orders considered by target D (which have no absorption contributions) by increasing escape cone diffraction outside this target (which does have some absorption benefit) might be favorable. The absence of this trend in QRD1-10 can be related to the fact that increased escape cone diffraction is hindered in this QR family, as all the lowest OS are already within the target. In that case, introducing more diffraction below the target’s lower boundary is not possible, even with poor localization.

For a given target, performance trends show that the QR families with the largest pitches have the most favorable performances. This observation is driven by the richer diffraction profiles of these structures, which result in higher contributions from diffracted power to the *J*_sc_ (see Figure S4). By populating more diffraction orders within the target, QR families with larger pitches can access more resonances of the richer modal structure of the transparent device concept across the spectrum. Although this indicates that performance improvements are possible upon increasing the pitch, *J*_sc_ seems to stabilize in the QR families with the largest pitches in targets B, C, and D. These comparable *J*_sc_ values are in fact a consequence of equivalent absorption profiles, which are smoothed due to the large collection of resonances (see Figure S4). Such comparable performance might indicate that increasing the amount of occupied diffraction orders in the target can eventually have diminishing returns. A finite number of incoupled photons and other loss mechanisms in the devices, such as transmission, reflection, or outcoupling, can prevent further performance improvements. However, this observation also indicates that the performance offered by transparent QR structures can be tolerant to pitch variations, provided low densities of optical states are avoided.

Finally, transparent QR structures provide the best performances when target spatial frequency ranges suppress evanescent losses and overlap predominantly with the device modal structure at the wavelengths that require absorption enhancement. To highlight this observation, representative device performances for the parameter combinations (QR family and spatial resolution) with best average *J*_sc_ for each target are shown in Figure 3f. Target D, where a significant amount of diffracted waves are evanescent close to the band gap and do not couple to the modal structure, shows the worst performance and the lowest contributions from diffracted power to the *J*_sc_. The thicker grating in this target (Table 2) increases resonance strength at long wavelengths to compensate for evanescent losses, but at the expense of absorption at shorter wavelengths. Similar results were observed in ref (25), where absorption enhancement in thicker 1 μm Si cells with QR structures was maximal when the target’s upper boundary extended to ∼20 μm^–1^, as beyond this frequency evanescent diffraction orders started to be populated at wavelengths near the absorber band gap. Transparent QR grating performance increases in target A, which avoids evanescent waves as well as escape cone losses. While target D also contained some escape cone losses below λ = 600 nm, this is not expected to be a main driver of its reduced performance. Targets B and C, which also contain equivalent or even greater escape cone losses, show the best performances with the highest contributions of diffracted power to the *J*_sc_ and no significant absorption drops at their threshold wavelength.

These observations suggest that transparent QR structures can achieve high efficiencies while being tolerant to escape cone losses. Targets A–C all have the same upper boundary that restricts evanescent losses, but cover progressively wider spatial frequency ranges by pushing the threshold wavelength closer to the band gap. As the target is widened, more wavelengths experience escape cone losses, but this also enables more waveguide modes to be accessed for wavelengths near the band gap that benefit the most from absorption enhancement due to their low absorption coefficients (see Figure 1c). To study this trade-off further, we design additional QR unit cells for two new targets, E and F, both with the same upper boundary (*k*_GaAs_ at λ = 900 nm) but with threshold wavelengths of 800 and 900 nm, respectively. Guided by our previous observations, these designs (with fill factor = 0.5, also included in Supporting Information, Discussion 4) have high spatial resolutions and large pitches for favorable performance (Table 1). The average performance of 10 unit cells for these new QR families (considering optimal ARC and grating thickness in Table 2) are shown in Figure 3e as a function of threshold wavelength. Representative results for other threshold wavelengths (QRA9-26, QRB9-50, and QRC9-68) taken from Figure 3a–c are also included. Transparent QR performance is shown to be remarkably stable upon increasing escape cone losses up to the absorber band gap, with minimal average reductions (0.29 mA/cm^2^) compared to the maximal performance (22.46 ± 0.9 mA/cm^2^) at λ_th_ = 700 nm. This tolerance to escape cone losses is likely due to the rich diffraction profile of these QR designs. Provided there is a high amount of occupied diffraction orders at *k*_*xy*_ where light trapping and waveguide mode coupling are available, losses by a few diffraction orders lying within the escape cone for wavelengths with incomplete absorption may not be detrimental.

To further support this argument, Figure 3e shows optimal performances of ultrathin devices with transparent photonic crystal gratings as a function of threshold wavelength (see Table S1 for optimal design parameters). The unit cells of these gratings have square SiO_2_ features in an Al_0.8_Ga_0.2_As matrix. As a result, their power spectral density will be similar to that in Figure 2a, with diffraction profiles highly localized at few diffraction orders and threshold wavelengths matching the pitch. Trends for such transparent photonic crystals are strikingly different to the QR designs. While optimal operation at λ_th_ = 577 nm achieves high device performance (22.91 mA/cm^2^), deviations in this optimal condition are highly detrimental to the point where for λ_th_ = 908 nm device performance drops by 3.16 mA/cm^2^. In photonic crystal gratings, the high localization of the diffraction profile at few diffraction orders leads to significant and prohibitive power losses whenever these are found within the escape cone.^22^

Finally, transparent QR structures can maintain high performances upon variations in the fill factor. Variations in this parameter were studied for the best performing parameter combination in Figure 3a–d (QRC5-37, *n* = 33, see Supporting Information Discussions 4 and 5), and average performances were found to stay above 22.5 mA/cm^2^ between fill factor values of 0.5–0.7. A fill factor of 0.6 yielded the highest *J*_sc_ at 22.86 ± 0.17 mA/cm^2^. This performance is equivalent to the best photonic crystal in Figure 3e, and in fact some QR devices within the studied population slightly outperform this structure. The fundamental advantage of transparent QR designs is their multiresonant light-trapping capability, which provides high performances together with resiliency toward design variation as needed to tolerate variability in fabrication processes.

## Weak Performance of Metallic QR Structures for Ultrathin Photovoltaics

**Figure 4:**
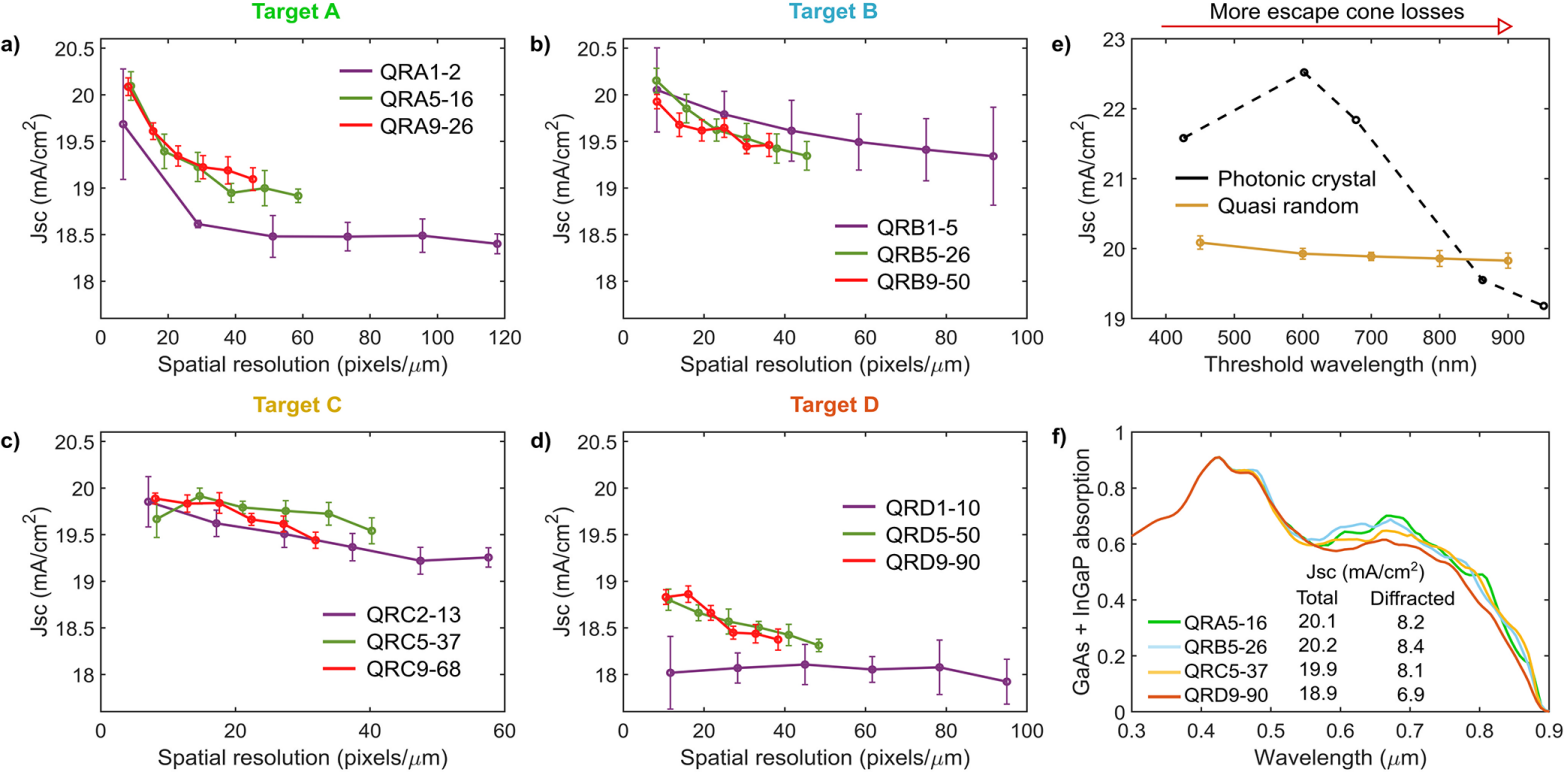
Light-trapping performance of metallic QR structures. (a–d) Average performance (*J*_sc_) of all 10 quasi-random unit cells designed for each target, QR family, and spatial resolution (matrix size), with the optimal parameters in Table 2 (error bars correspond to the standard deviation of the *J*_sc_ for a given population). (e) Performance of metallic QR structures and optimal photonic crystals as a function of the threshold wavelength, which marks the spectral limit below which power is diffracted within the escape cone. (f) Absorption (GaAs + InGaP) plots for representative devices of the best performing QR family and resolution for each target.

The performance of QR gratings for different targets, pitches (QR families) and matrix sizes (spatial resolutions) is shown in Figure 4a–d for metallic devices. Only average performances are shown for all 10 unit cells of each parameter combination. Error bars indicate performance deviations within this population, which are low in most cases (generally <0.3 mA/cm^2^).

Metallic QR gratings provide drastically reduced performances in ultrathin devices compared to transparent structures. Key differences between these designs are the reduced modal structures offered by the metallic grating together with the parasitic absorption in the metallic texture. Average losses in the metallic QR gratings stayed above 5 mA/cm^2^ in all cases and reached up to 9 mA/cm^2^ (see Figures S5 and S6).

The optical losses in metallic QR gratings have an impact on spatial resolution trends, causing device performance to decrease upon increasing this parameter. For almost all QR families and contrary to the transparent case, higher feature resolutions lead to a drop in device performance that can approach 1 mA/cm^2^ within the resolution range studied. This reduction in the *J*_sc_ is related to higher power losses in the grating as resolution is increased. As discussed in Supporting Information, Discussion 6, a possible mechanism behind this observation is the increase in metallic discontinuities found in textures with higher resolutions, which can enhance field interactions with the Ag and lead to greater power losses. Overall, performance trends highlight limited tolerance to spatial resolution variations in metallic QR designs.

Contrary to the transparent case, integrating metallic QR gratings with larger pitches does not necessarily lead to better device performances (see also Figure S7). Although higher performances can be observed in targets A and D for larger pitches, no clear trends are found in targets B and C, where different QR families have equivalent performances. These observations may be related to the reduced modal structure in metallic device concepts. Introducing more diffraction orders by increasing the pitch may not result in a significant increase of waveguide resonances if the modal structure is restricted and power dissipation in the modes is high due to low quality factors. Although these observations do not indicate that performance stability upon varying the pitch is significantly compromised in metallic designs, they do expose the limited gains that are available by changing this parameter.

Finally, as observed with transparent designs, metallic QR gratings offer the best device performances when spatial frequency targets avoid evanescent losses and have significant overlap with the device modal structure at the wavelengths that need absorption enhancement. Figure 4f shows representative device performances for the parameter combinations (QR family and spatial resolution) with the best average *J*_sc_ for each target. Target D populates evanescent diffraction orders and is shown to have the worst performance as well as the lowest contributions from diffracted waves to the *J*_sc_. The other targets restrict evanescent diffraction and have increased performances, although that of target C is slightly reduced compared to A and B. This reduction is associated with weaker diffraction contributions to the *J*_sc_. Among A, B, and C, target C covers the widest spatial frequency range and has the largest threshold wavelength leading to more escape cone losses. Although in transparent devices it was suggested that these losses could be compensated by accessing more waveguide modes near the band gap, in metallic devices this mechanism is expected to be weaker considering the reduced number of waveguide modes that are accessible. Weaker compensation of escape cone losses would make these more detrimental and result in device performance peaking at shorter λ_th_.

To further study this proposition, we design additional QR unit cells for targets E (λ_th_ = 800 nm) and F (λ_th_ = 900 nm), all with a fill factor of 0.5 (Supporting Information, Discussion 4). Contrary to the transparent case, this time the designs have large pitches but the lowest possible resolutions in order to have the most favorable performance (Table 1). The average *J*_sc_ of 10 unit cell designs (considering optimal ARC and grating thicknesses in Table 2) for these targets is shown in Figure 4e, together with representative results of gratings with different threshold wavelengths (QRA9-26, QRB9-50, and QRC9-68) that also have the lowest resolutions and large pitches. As observed in transparent designs, the performance of metallic QR gratings does not vary significantly as escape cone losses are increased, although it does seem to peak at shorter threshold wavelengths. This observation suggests that although metallic QR designs can have weaker compensation of escape cone losses, these are still not highly detrimental. Given the rich diffraction profile of these structures, the fraction of power diffracted into the escape cone is expected to be reduced if the majority of occupied diffraction orders lie above *k*_0_.

As with our analysis of the transparent QR gratings, we further study the relationship between diffraction profile and tolerance to escape cone losses by designing and evaluating metallic photonic crystal gratings for different threshold wavelengths. These gratings have the same geometry as in the transparent case, but this time, SiO_2_ features are found in a Ag matrix. The performance of these metallic photonic crystal gratings with optimal parameters (Table S2) is found in Figure 4e. As with transparent designs, the performance of metallic photonic crystal gratings is severely affected by escape cone losses due to their highly localized diffraction profiles. Performance drops by 3.34 mA/cm^2^ from the best condition at λ_th_ = 602 nm to the lowest performance at λ_th_ = 952 nm. However, the best performing metallic photonic crystal has a high *J*_sc_ (22.52 mA/cm^2^), considerably better than any of the metallic QR structures and close to that of optimal transparent QR and photonic crystal gratings. The origin of this pronounced difference will be discussed in the next section.

Finally, metallic QR designs can have stable performances upon varying the fill factor. We studied changes in this parameter for the best performing parameter combination of Figure 4a–d (QRB5-26, *n* = 11, see Supporting Information, Discussions 4 and 5). Average performances stayed above 19.8 mA/cm^2^ in the range from 0.4–0.6, with the best *J*_sc_ (20.15 ± 0.13 mA/cm^2^) found at a fill factor of 0.5. This performance is significantly lower than that of the best transparent QR design by almost 3 mA/cm^2^. Overall, metallic QR structures offer limited benefits to ultrathin solar cells, with main drivers expected to be their higher parasitic absorption and reduced modal structures. If a metal is to be used for a scattering structure in an ultrathin solar cell, a photonic crystal geometry is much more beneficial, although care must be taken to meet the stringent design requirements for optimal operation.

To conclude, we highlight that our findings unify previous conclusions on the light-trapping benefits of “quasi-periodic” or QR textures in ultrathin devices. Studies that reported limited absorption enhancement implemented metallic features with reduced modal structure, high resolution and weak modal overlap.^32,33^ On the other hand, the works that reported favorable light trapping implemented dielectric/semiconductor textures that enhanced the modal structure, avoided low resolutions, enabled high densities of optical states and had predominant modal overlap restricting evanescent diffraction.^24,31,34,38^ Note that in some of these cases the texture cannot be classified as transparent as the semiconductor in this layer also functions as the absorber material. However, in such cases the absorption in this texture is not parasitic and high performances remain achievable.

## Light-Trapping Mechanisms in Ultrathin Devices with Photonic Crystal and QR Scattering Structures

**Figure 5:**
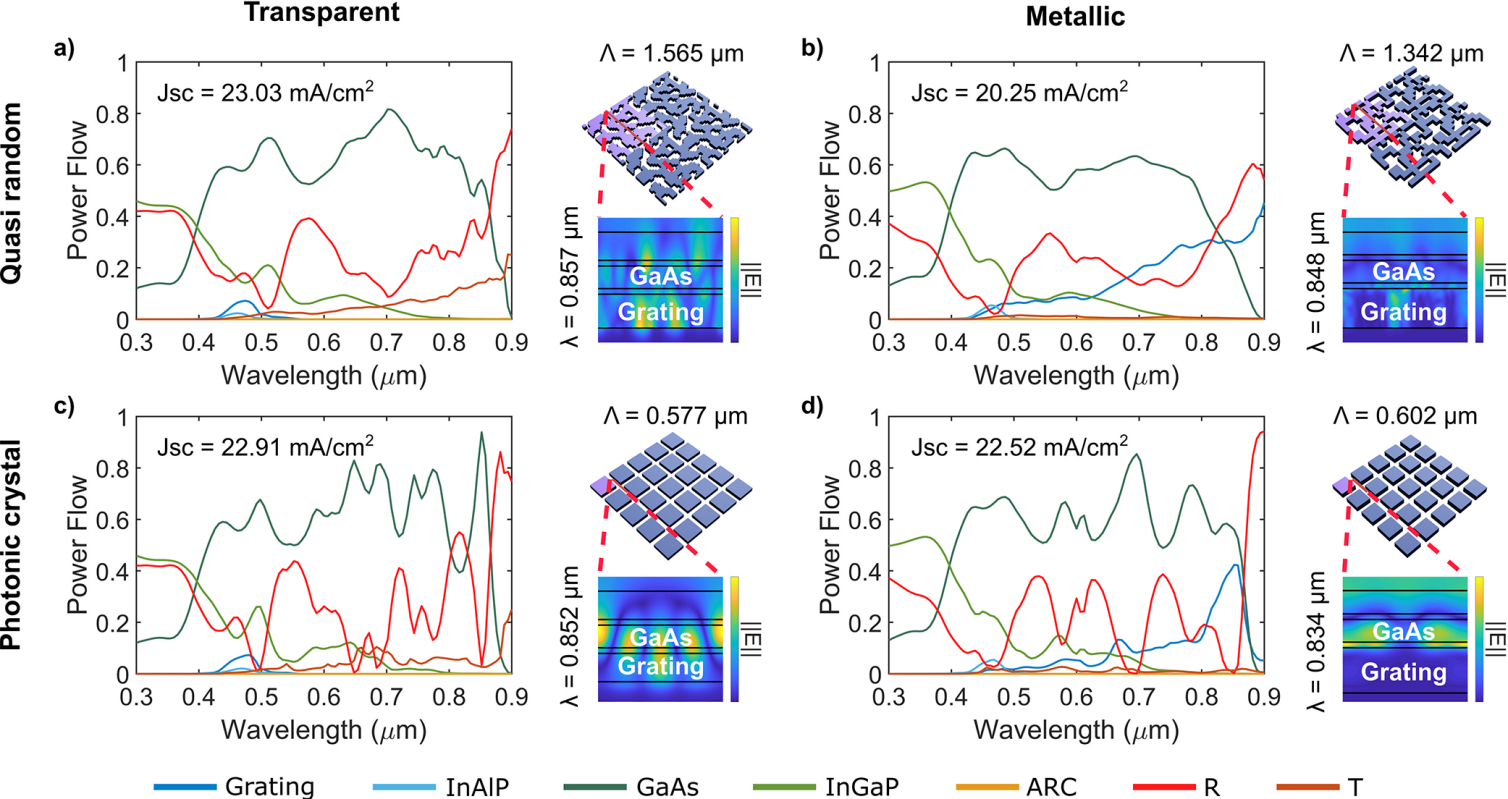
Best performing photonic crystal and QR structures for metallic and transparent devices. Cross-sectional field profiles are obtained for s polarized light at wavelengths where a coupling event is present near the band gap to the lowest order waveguide mode for TE polarization. All designs only show dielectric features and highlight those within one unit cell. Solid red lines on unit cells mark the *xy* coordinates at which the field profiles are calculated.

To highlight relevant light-trapping mechanisms in ultrathin solar cells with different scattering structures, we make a comparison of the absorption profiles of optimal transparent and metallic designs for both QR and photonic crystal gratings (Figure 5).

Ultrathin solar cells with either metallic or transparent photonic crystal gratings show clear absorption peaks in the GaAs layer. Features below λ = 500 nm are due to thin-film effects, affected by grating and ARC thicknesses. Waveguide resonances appear at longer wavelengths. The highly localized power spectral density and diffraction profile of photonic crystals leads to strong coupling to waveguide modes but limits the number of resonances in the spectrum. Due to their modal structures, the GaAs absorption peaks in the metallic case are broader and more scarce than those in the transparent design. The performance of the transparent photonic crystal is higher, but that of the metallic design is comparable given strong scattering of the metallic texture and the fact that its broader resonances can partly compensate for the reduced absorption at off-resonance conditions. Another important mechanism leading to a favorable performance with the metallic photonic crystal grating is its strong coupling of incident light to the modal structure. When coupled to a waveguide mode, the field can be localized within the active layer and away from the metallic texture where parasitic absorption would take place. This localization within the absorber is enabled by interference of the propagating waves in the mode. An example of such localization is shown in the representative field profile of Figure 5d for the metallic photonic crystal case, obtained at a waveguide resonance close to λ = 850 nm. High localization within the active layer can also occur with transparent photonic crystal gratings as shown in the representative field profile of the transparent photonic crystal (Figure 5c), also obtained at a waveguide resonance near the band gap.

On the other hand, ultrathin devices with either metallic or transparent QR gratings show smoother absorption spectra in the GaAs due to the ability of QR designs to introduce a collection of resonances throughout the spectrum (Figure 5 a-b). As highlighted in the previous section, the performance of the metallic QR structure is drastically reduced compared to the metallic photonic crystal grating. This difference may be related to the increased number of metallic discontinuities in the QR designs, but is also expected to be a consequence of the broader scattering profile of these structures. QR gratings have weaker coupling to waveguide modes, which can lead to lower field localization within the active layer and higher parasitic absorption in the metallic texture. Supporting this argument, the absorption in the light-trapping layer of the metallic QR design in Figure 5b is high and significantly exceeds that of the metallic photonic crystal grating in Figure 5d. Similar field localization mechanisms are expected when employing transparent QR gratings, but in such cases the field interactions with the light-trapping layer do not lead to parasitic losses and so these designs can still reach high performances that compete with photonic crystals. This discussion is further supported by the representative field profiles for both metallic and transparent QR gratings shown in Figure 5a,b. These are obtained at wavelengths close to the band gap where waveguide resonances are expected given device modal structures, and show a considerably more delocalized field than the one in the devices with photonic crystals.

Finally, although the performance of the optimal transparent QR structure is high, its absorption profile reveals that further improvements to light harvesting are possible by minimizing reflection losses. Such improvements are possible via the integration of more sophisticated antireflection strategies than the simple ARC studied in this work. For example, replacing the top SiO_2_ layer in the device studied in Figure 5a with a double-layer ARC (85 nm MgF_2_/50 nm Ta_2_O_5_) can further boost the *J*_sc_ and reach 24.24 mA/cm^2^ without changing the rest of the device architecture. This *J*_sc_ represents an improvement of 43% compared to an equivalent planar device at 16.94 mA/cm^2^ with an optimal 60 nm MgF_2_/35 nm Ta_2_O_5_ double-layer ARC (see Figure S8 for a comparison of the absorption profiles of these devices). Considering the electrical performance that is achievable with our device architecture of interest, we estimate that transparent QR gratings hold the potential to reach up to 20% efficiency with an 80 nm GaAs absorber (see Supporting Information, Discussion 7). This remarkable performance on such a thin length-scale represents one of the highest absorption enhancements reported to date for textured GaAs solar cells (see Supporting Information, Discussion 8) and is unlocked by a scattering structure that holds unique engineering tolerance. Further improvements are possible, for example, by using rear spacers to minimize losses in the Ag mirror^31^ or by employing transparent grating materials with even more beneficial index contrast for light trapping.^35^

## Conclusions

New light-trapping platforms are needed to boost photovoltaic efficiencies in ultrathin solar cells where absorption enhancement can be limited by a reduced number of waveguide modes. Integrating scattering layers made with transparent semiconductor/dielectric materials is demonstrated to be a compelling light-trapping strategy that enriches the modal structure compared to metallic textures, offering more resonances and minimizing parasitic losses. These properties allow engineered quasi-random scattering structures to achieve high efficiencies in ultrathin solar cells when designed with transparent materials, given that their broad scattering profile is well-suited to couple to a rich modal structure and achieve broadband absorption enhancement. Metallic quasi-random scatterers, however, do not provide favorable performance in ultrathin devices, as they suffer from limited resonances together with high absorption losses in the metal. To exploit the light-trapping benefits of transparent quasi-random structures, we have provided thorough guidelines for an optimal design following an exhaustive exploration of the available design space. Main requirements for favorable performance with transparent quasi-random structures are restricting evanescent losses, avoiding low feature resolutions and enabling high densities of optical states where the occupied diffraction orders predominantly overlap with the modal structure. Provided these conditions are met, high performances in ultrathin device stacks are achieved with remarkable tolerance to design variability and escape cone losses. Although transparent photonic crystal gratings can offer comparably high performances, the key advantage of transparent QR designs is their ability to relax the conditions needed to achieve optimal operation. Overall, the integration of transparent QR structures is exposed as a viable strategy to push light harvesting in the thinnest length-scales, one that is available for a wide range of material systems and device architectures, and compatible with scalable low-cost fabrication techniques due to its tolerance to process variability.

## Methods

### Waveguide Analysis

The propagation constants of waveguide modes for different devices were calculated with a transfer matrix method (TMM).^22^ The TMM considers the solar cell as a stack of planar layers, each having a certain thickness and complex refractive index. Scattering structures are treated as uniform media, with effective refractive indices defined by the weighted average of the optical constants of the materials in the unit cell (according to the fill factor). The TMM obtains dispersion equations whose roots correspond to the waveguide modes available for a given wavelength and polarization of light. These roots are solved with a Newton–Raphson method in the complex plane, looking in the range between *k*_0_–*k*_GaAs_ (real) for β′ and for β″ values satisfying β′/β″ (quality factor^37^) > 3.

### Rigorous Coupled-Wave Analysis Simulations

Absorption simulations were done with GD-Calc.^39^ Results are reported as averages of s and p polarization. A rectangular truncation scheme was followed to define Fourier orders, the number of which was selected for different QR families according to the optical states within their corresponding target (based on convergence studies, available in Supporting Information, Discussion 9). For photonic crystal unit cells, 225 Fourier orders were considered.

Field profiles were obtained with RayFlare^40^ using a modified version of the RCWA implementation *S*^4^.^41^ A circular truncation scheme was used, with 70 Fourier orders for photonic crystal structures and 300 Fourier orders for QR structures. Equivalent peaks in both RCWA implementations (GD-Calc and *S*^4^) were seen at the studied wavelengths.

### Optical Constants

The optical constants of the GaAs, InGaP, and InAlP layers are those found in ref (21). Those of Ag were taken from ref (42), whereas those of Al_0.8_Ga_0.2_As were digitized from ref (43). Values for Ta_2_O_5_ were taken from ref (44), and those of MgF_2_ were taken from ref (45). SiO_2_ was considered as a lossless, dispersionless material with *n* = 1.46. These optical constants were used for all RCWA and TMM optical simulations.

### Design Algorithm for QR Structures

To design a QR unit cell for a given QR family, an initial seed is created that consists of a random *n* × *n* array with the appropriate fill factor and size (*n* is an odd number). Entries in this array are 1s and 0s representing two different grating materials. 1s are assigned to SiO_2_ in all cases, whereas 0s are assigned to either Ag or Al_0.8_Ga_0.2_As, depending on the device concept under consideration. After defining the initial seed, two opposite entries in the array are then randomly selected and swapped if this reduces the following optimization target *T*:
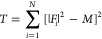
3where *F*_*i*_ is the Fourier amplitude (calculated with a Fast Fourier Transform algorithm) at an optical state *i* within the target region under consideration (for a given pitch and QR family), *N* is the total number of optical states within this target, and *M* is given by the following equation:
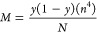
4where *y* is the fill factor and *n* is the size of the *n* × *n* array representing the unit cell (the derivation of the optimization target *T* is included in Supporting Information, Discussion 3). This process occurs for a certain number of iterations before the design is finalised. The number of iterations (in the order of 10^4^–10^5^) was different for every QR family and resolution, and was selected based on the convergence of the target *T* for the particular design under consideration.

For the designs with fill factor different than 0.5 as well as those of target F, the output of the previously described process was used as the seed for another algorithm for further optimization. In this algorithm, a random entry in the array is selected and swapped with a neighboring entry (up, down, left, or right) if this reduces the target *T* further. This process occurred for 5 × 10^4^ iterations in all cases.

## Supporting Information Available

Optical constants of grating materials; absorption vs pitch scans for ordered photonic crystals; flowchart depicting the different stages of the design space study; representative absorption profiles; losses in QR gratings; absorption spectra of optimal QR and planar devices; optimal parameters for photonic crystals at different threshold wavelengths; correlation of absorption peaks to waveguide modes; localization of the power spectral density in QR unit cells; derivation of target for QR design; QR unit cells; fill factor studies; discontinuities and losses in QR designs; estimation of achievable photovoltaic efficiency; state-of-the-art performances for GaAs solar cells; convergence studies (PDF)
