# Supplementary material for: Transparent Quasi-Random Structures for Multimodal Light Trapping in Ultrathin Solar Cells with Broad Engineering Tolerance
Source: ACS Photonics. 2022 Jun 23;9(8):2724–35. doi: 10.1021/acsphotonics.2c00472 (PMC9389614; doi:10.1021/acsphotonics.2c00472)
Supplement: Supplementary file 1 — ph2c00472_si_001.pdf [file ph2c00472_si_001.pdf]

Supporting information for "Transparent quasi-random structures for multi-modal light trapping in ultra-thin solar cells with broad engineering tolerance"

Eduardo Camarillo Abad[1], Hannah J. Joyce[2], and Louise C. Hirst[1,3]

[1] *Department of Physics, University of Cambridge, Cambridge, UK*
[2] *Department of Engineering, University of Cambridge, Cambridge, UK*
[3] *Department of Materials Science and Metallurgy, University of Cambridge, Cambridge, UK*

Contents

Supplementary figures S2
Supplementary tables S10
Supplementary Discussion 1: Comparison of TMM and RCWA resonant pitches for representative devices with transparent and metallic light trapping layers S12
Supplementary Discussion 2: Power spectral density localisation in different QR unit cells S14
Supplementary Discussion 3: Derivation of QR optimisation target S15
Supplementary Discussion 4: QR unit cell designs S16
Supplementary Discussion 5: Fill factor studies in QR designs S31
Supplementary Discussion 6: Effect of increased spatial resolution on the parasitic losses in metallic QR textures S32
Supplementary Discussion 7: Estimation of achievable photovoltaic efficiency with optimal transparent QR structures S34
Supplementary Discussion 8: State-of-the-art performances for thin and ultra-thin GaAs solar cells S35
Supplementary Discussion 9: Convergence of RCWA simulations of QR structures S37

Supplementary figures

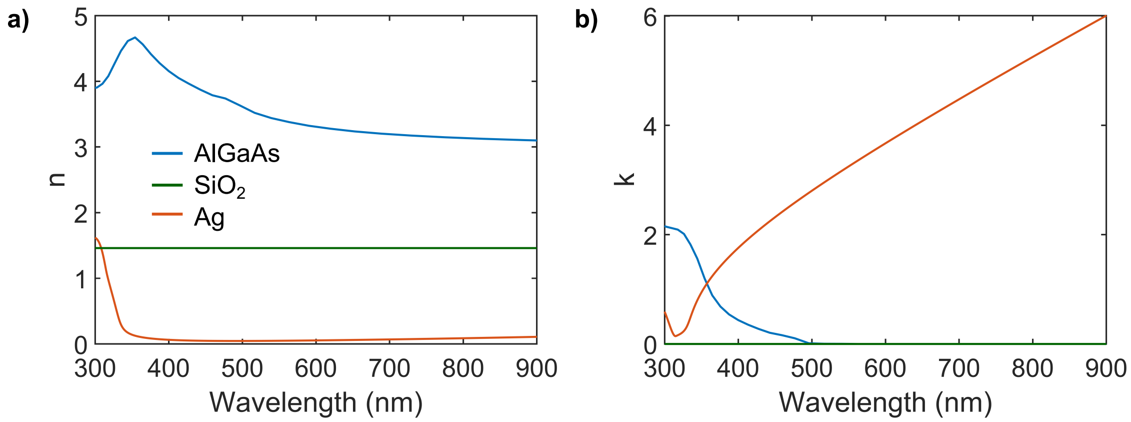

Figure S1: **Optical constants of grating materials.** Optical constants n (a) and k (b) of the materials considered for metallic and transparent light trapping layers. Sources of the optical constants are included in Methods (main text).

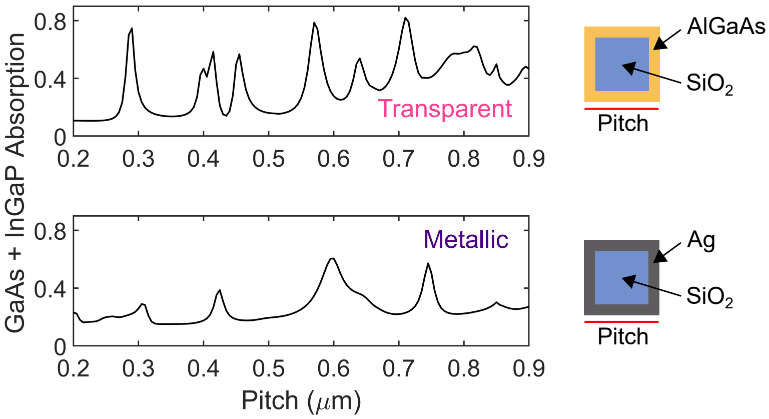

Figure S2: **Revealing resonances in ultra-thin solar cells with metallic and transparent photonic crystal textures.** Absorption as a function of pitch ($\lambda = 850$ nm) in the GaAs and InGaP layers of the device architecture of interest for the case of transparent and metallic photonic crystal gratings (ARC thickness = 100 nm, grating thickness = 100 nm and fill factor = 0.5 in both cases). Grating unit cells are shown with the corresponding material distributions. Transparent photonic crystal gratings support more abundant and stronger resonances than the metallic case.

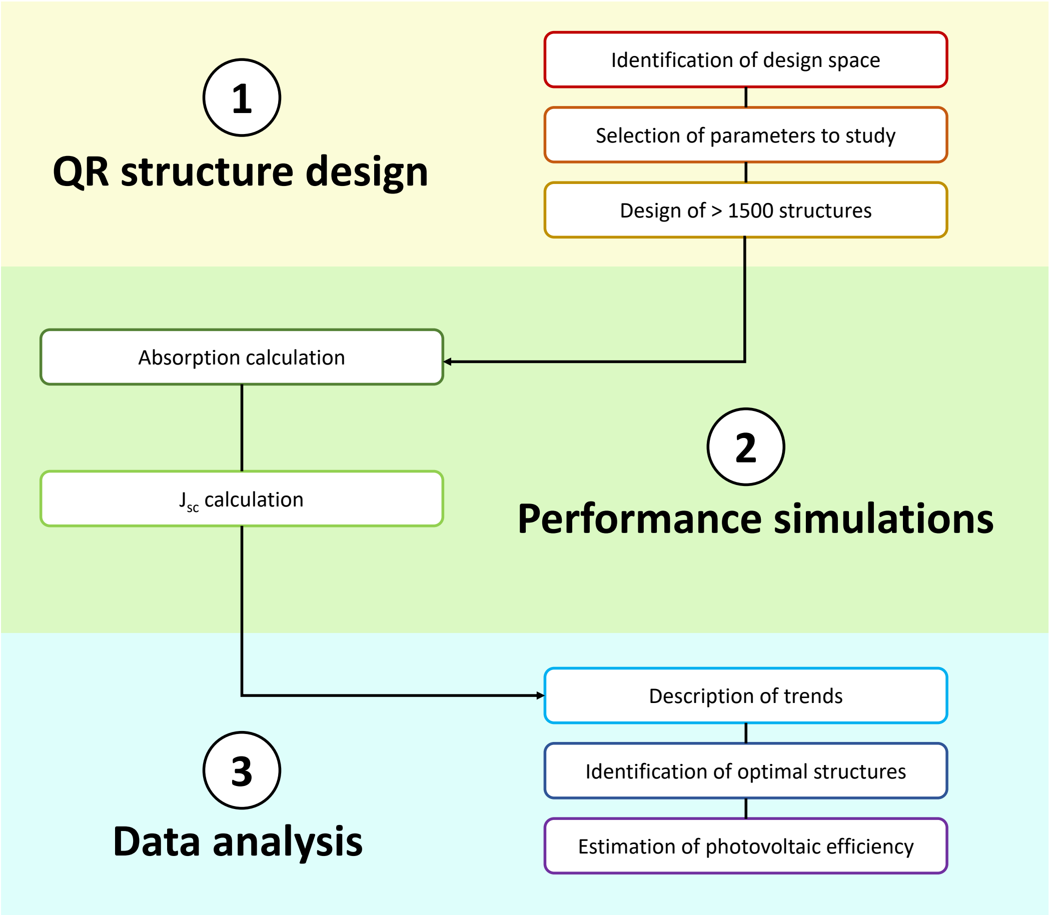

Figure S3: **Flowchart of our QR light trapping study.** Our exploration of the performance of different QR designs in ultra-thin GaAs solar cells comprised three main steps: design of QR structures, performance simulations, and data analysis.

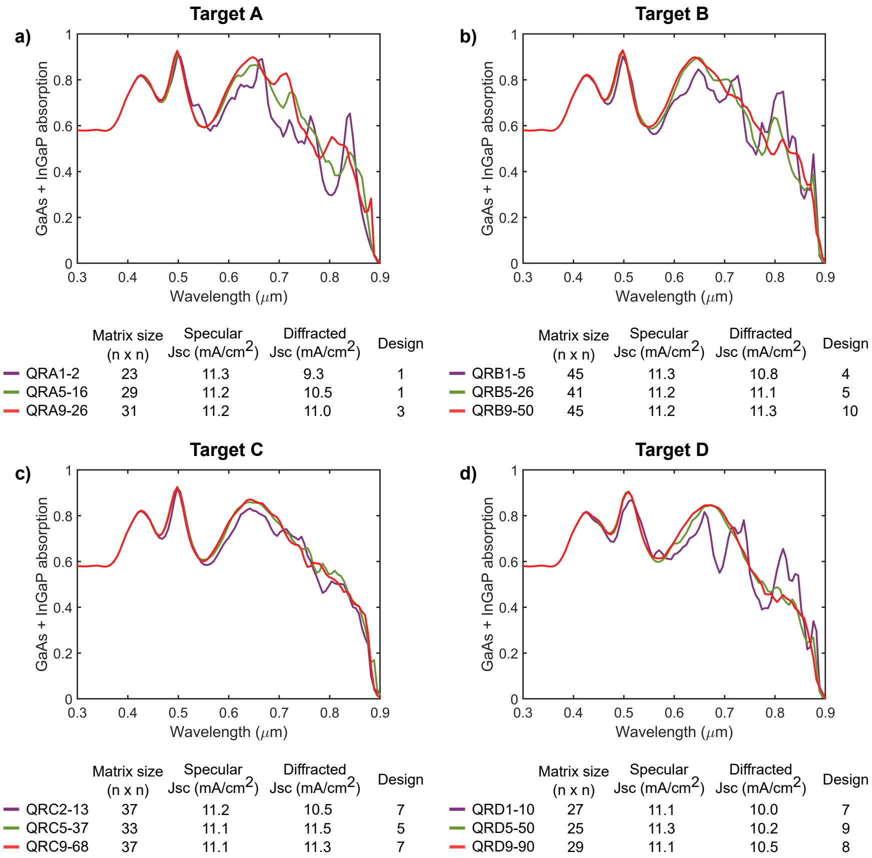

Figure S4: **Representative absorption profiles of devices with transparent QR gratings.** Absorption in the GaAs and InGaP layers as a function of wavelength for representative devices of each QR family and targets A (a), B (b), C (c) and D (d) (fill factor 0.5), with the optimal parameters in Table 2 (main text). Also included are the contributions of specular and diffracted waves to the Jsc. Design number corresponds to those in Supplementary Discussion 4.

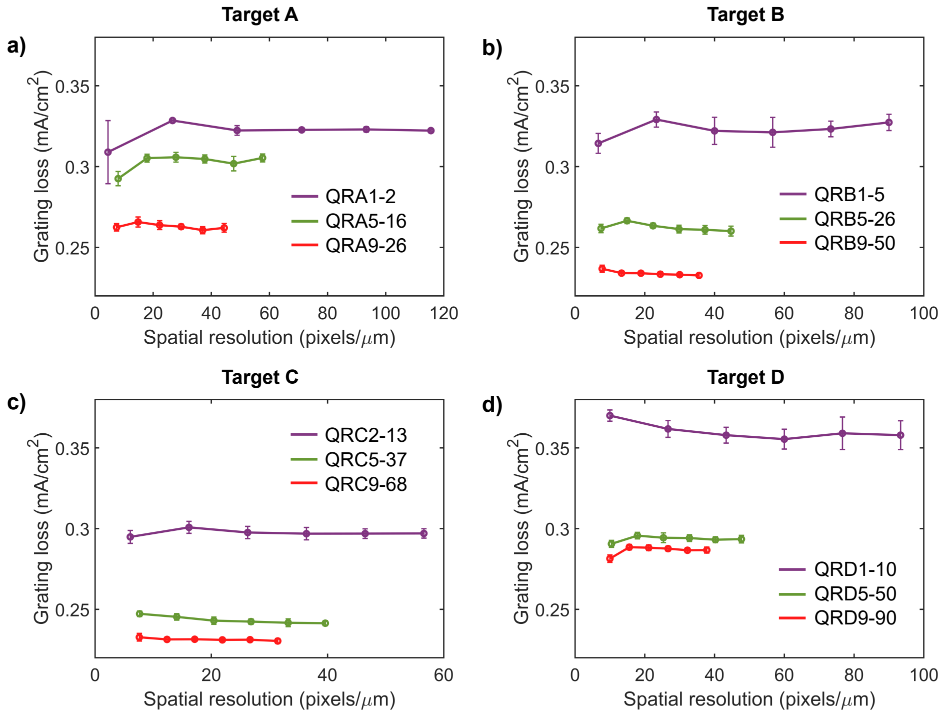

Figure S5: **Losses in transparent QR textures.** Average parasitic loss of all 10 quasi-random gratings designed for different QR families and spatial resolutions (matrix size) in targets A (a), B (b), C (c) and D (d) at fill factor 0.5, considering transparent textures. Losses are reported as the photocurrent that corresponds to the absorption in the light trapping layer. Simulations consider the optimal parameters in Table 2 (main text).

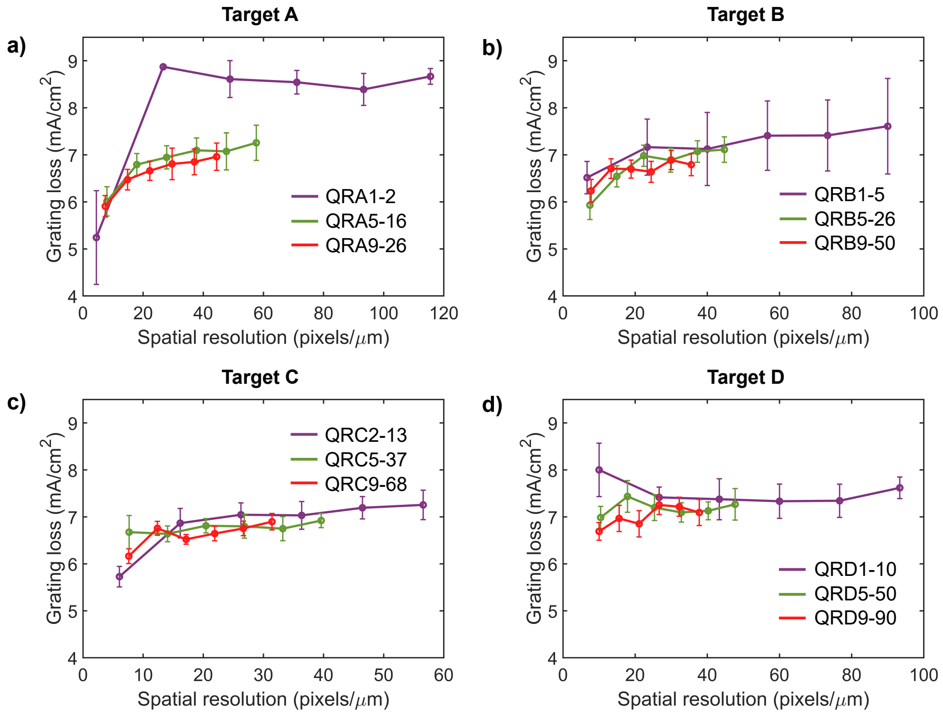

Figure S6: **Losses in metallic QR textures.** Average parasitic loss of all 10 quasi-random gratings designed for different QR families and spatial resolutions (matrix size) in targets A (a), B (b), C (c) and D (d) at fill factor 0.5, considering metallic textures. Losses are reported as the photocurrent that corresponds to the absorption in the light trapping layer. Simulations consider the optimal parameters in Table 2 (main text).

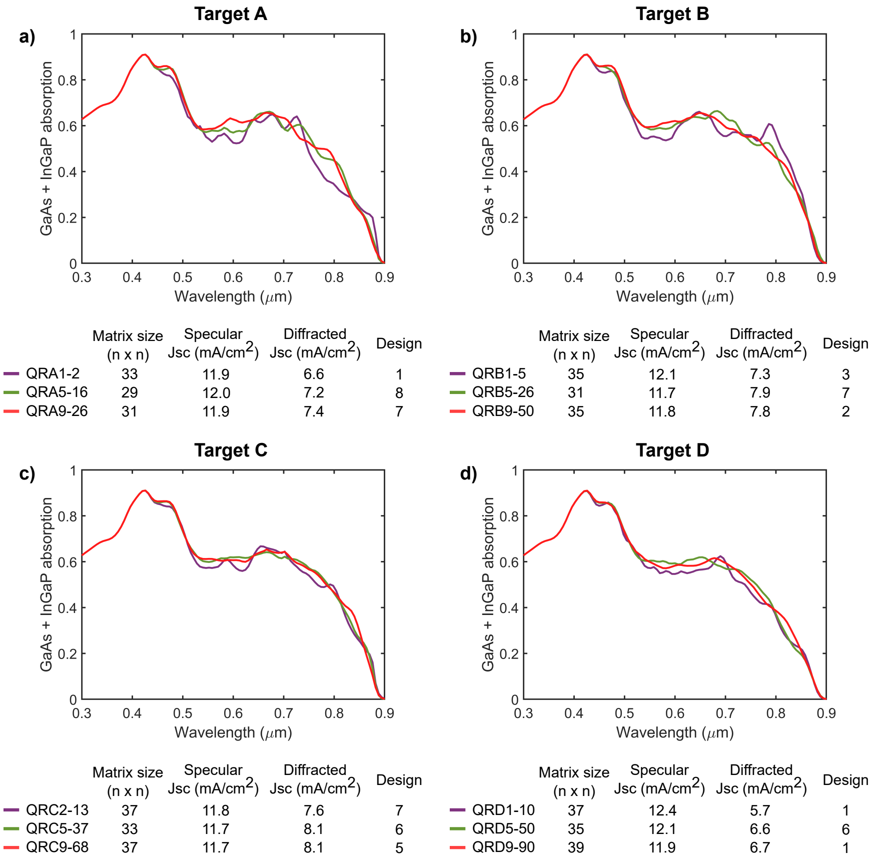

	Matrix size (n x n)	Specular Jsc (mA/cm^2)	Diffracted Jsc (mA/cm^2)	Design
QRA1-2	33	11.9	6.6	1
QRA5-16	29	12.0	7.2	8
QRA9-26	31	11.9	7.4	7

	Matrix size (n x n)	Specular Jsc (mA/cm^2)	Diffracted Jsc (mA/cm^2)	Design
QRB1-5	35	12.1	7.3	3
QRB5-26	31	11.7	7.9	7
QRB9-50	35	11.8	7.8	2

	Matrix size (n x n)	Specular Jsc (mA/cm^2)	Diffracted Jsc (mA/cm^2)	Design
QRC2-13	37	11.8	7.6	7
QRC5-37	33	11.7	8.1	6
QRC9-68	37	11.7	8.1	5

	Matrix size (n x n)	Specular Jsc (mA/cm^2)	Diffracted Jsc (mA/cm^2)	Design
QRD1-10	37	12.4	5.7	1
QRD5-50	35	12.1	6.6	6
QRD9-90	39	11.9	6.7	1

Figure S7: **Representative absorption profiles of devices with metallic QR gratings.** Absorption in the GaAs and InGaP layers as a function of wavelength for representative devices of each QR family and targets A (a), B (b), C (c) and D (d) (fill factor 0.5), with the optimal parameters in Table 2 (main text). Also included are the contributions of specular and diffracted waves to the Jsc. Design number corresponds to those in Supplementary Discussion 4.

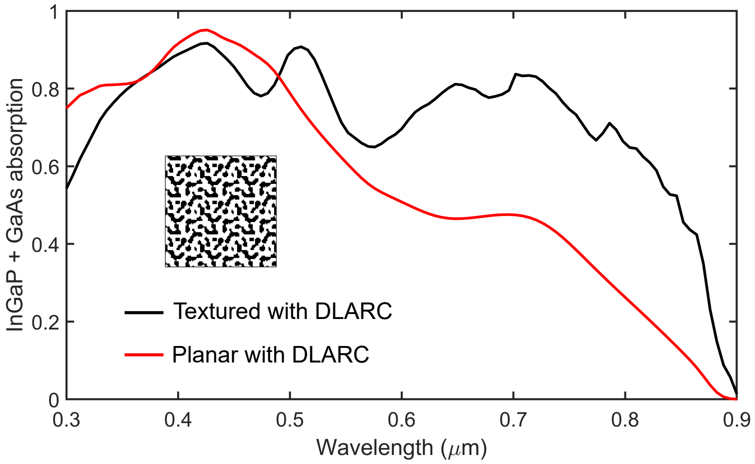

Figure S8: **Absorption profiles of optimal QR and planar devices with double-layer antireflection coating.** Simulated absorption in the InGaP and GaAs layers of optimal textured and planar ultra-thin GaAs solar cells. Device architecture corresponds to the one shown in Fig. 1 a (main text), except that a double-layer MgF_2/Ta_2O_5 antireflection coating (DLARC) is implemented instead of a single layer SiO_2 antireflection coating. Optimal thicknesses for the DLARC in the planar device are 60 nm MgF_2/35 nm Ta_2O_5, whereas those for the textured device are 85 nm MgF_2/50 nm Ta_2O_5. The QR design for the textured case is shown as inset (white areas correspond to SiO_2 and black areas to $Al_{0.8}Ga_{0.2}As$), and has a thickness of 120 nm.

Supplementary tables

Table S1: Optimal design parameters for transparent photonic crystals in ultra-thin devices

Threshold wavelength (nm)	Fill factor	Grating thickness (nm)	ARC thickness (nm)
407	0.5	100	100
577	0.6	100	100
638	0.4	120	100
817	0.6	100	100
908	0.5	120	80

Table S2: Optimal design parameters for metallic photonic crystals in ultra-thin devices

Threshold wavelength (nm)	Fill factor	Grating thickness (nm)	ARC thickness (nm)
426	0.5	180	80
602	0.5	160	80
678	0.4	160	80
863	0.3	160	80
952	0.5	160	80

Supplementary Discussion 1: Comparison of TMM and RCWA resonant pitches for representative devices with transparent and metallic light trapping layers

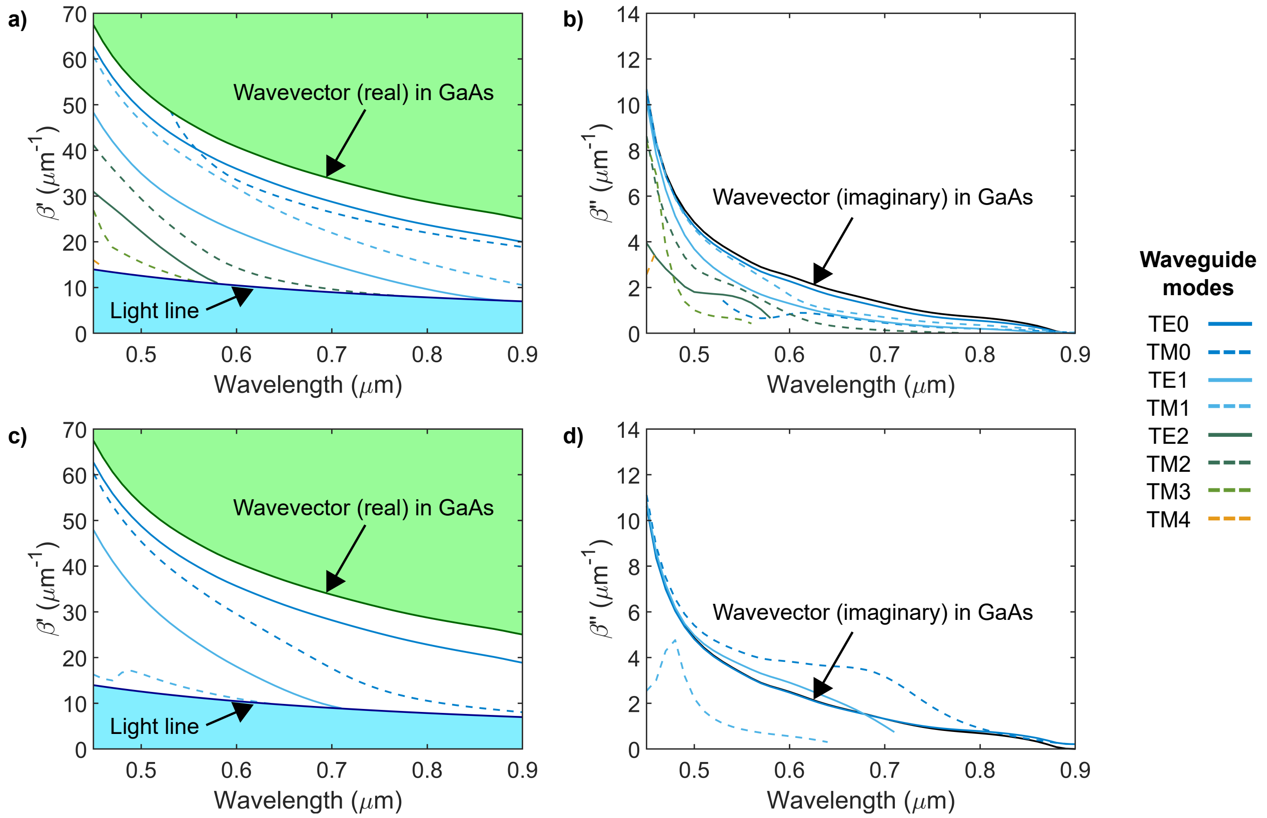

Figure S9: a) Real and b) imaginary propagation constants of the waveguide modes supported by the representative transparent devices studied in Fig. 1 c and d in the main text. c) Real and d) imaginary propagation constants of the waveguide modes supported by the representative metallic devices studied in Fig. 1 c and d in the main text.

The real (β') and imaginary (β'') waveguide mode propagation constants calculated for representative devices with metallic and transparent light trapping layers, shown in Fig. 1 c and d in the main text, are replotted in Fig. S9. Normally-incident light on the devices will be able to couple to one of these modes whenever the pitch matches the in-plane spatial frequency of a set of diffracted orders (k_{xy}, defined by the pitch via equation 1 in the main text) to the real propagation constant β' of the corresponding mode. We calculate the 'resonant pitches' at which such phase matching condition would be met for the different waveguide modes in Fig. S9 at different sets of diffracted orders (OS). Knowledge of the resonant pitches then allows us to attribute the absorption peaks in Fig. 1 e (main text) to different waveguiding events at different OS. Tables S3 and S4 contain these attributions for the transparent and metallic cases, respectively.

Table S3: Modal analysis for transparent light trapping layer

RCWA peak location (μm)	Attribution and calculated resonant pitch (μm)
0.290	TE0@OS1 (0.288) and TM0@OS1 (0.309)
0.405	TE0@OS2 (0.407)
0.460	TM0@OS2 (0.437)
0.480	TM1@OS1 (0.493)
0.570	TE0@OS4 (0.575)
0.605	TM0@OS4 (0.618)
0.645	TE0@OS5 (0.643)
0.695	TM0@OS5 (0.691) and TM1@OS2 (0.697)
0.800	TE1@OS1 (0.805)
0.825	TE0@OS8 (0.813)
0.850	TE0@OS9 (0.863) and TM0@OS8 (0.874)

Table S4: Modal analysis for metallic light trapping layer

RCWA peak location (μm)	Attribution and calculated resonant pitch (μm)
0.300	TE0@OS1 (0.301)
0.435	TE0@OS2 (0.426)
0.610	TE0@OS4 (0.602)
0.650	TE0@OS5 (0.673)
0.850	TE0@OS8 (0.851)

Supplementary Discussion 2: Power spectral density localisation in different QR unit cells

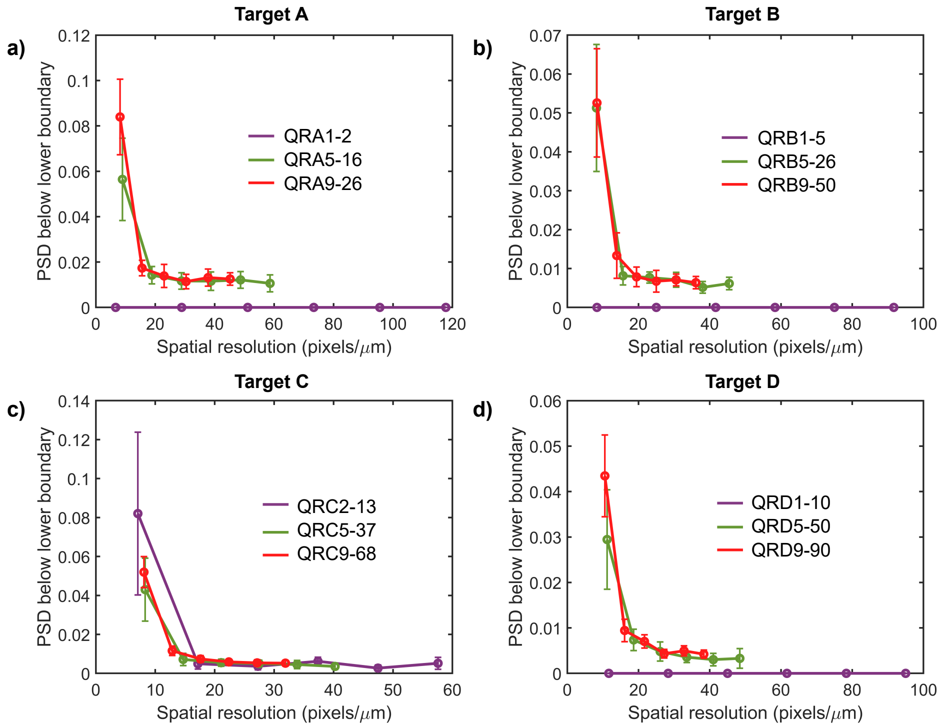

Figure S10: Mean nonspecular power spectral density (PSD) below the lower boundary of the corresponding target of all 10 QR unit cells designed for different targets (A-D), QR families and spatial resolutions at fill factor 0.5. Data are normalised by the total nonspecular power spectral density.

Fig. S10 shows the mean nonspecular power spectral density (PSD) below the lower boundary of the corresponding target of all 10 unit cells designed for different targets (A-D), QR families and spatial resolutions (at fill factor 0.5). Data were calculated by obtaining the Fast Fourier Transform of a particular unit cell design, calculating the power spectral density and then adding up all the values corresponding to spatial frequencies below the lower boundary of the target (without considering the zero frequency component OS0). The data were then normalised by the total nonspecular power spectral density. For QR families with OS1 at the lower boundary of the target (QRA1-2, QRB1-5 and QRD1-10), there are no nonspecular optical states below the target's lower boundary (since OS1 is at the lower boundary and is the lowest OS after OS0), and so the PSD below this lower boundary is always zero.

Supplementary Discussion 3: Derivation of QR optimisation target

From Parseval's theorem we have the following expression:

$$\sum_{i=0}^{n-1}\sum_{j=0}^{n-1}|f(i,j)|^2 = \frac{1}{n^2}\sum_{k=0}^{n-1}\sum_{l=0}^{n-1}|X(k,l)|^2 \tag{1}$$

where $f(i,j)$ is an entry in the n x n unit cell array and $X(k,l)$ is an entry in its corresponding Fast Fourier Transform. Considering that, for our arrays of 1s and 0s, $\sum_{i=0}^{n-1}\sum_{j=0}^{n-1}|f(i,j)|^2$ is defined by the fill factor y as yn^2, and that the entry in the Fast Fourier Transform corresponding to the zero frequency component (the 'specular' component) is equal to yn^2, the following equation can be written for the nonspecular power spectral density PSD (the result of $\sum_{k=0}^{n-1}\sum_{l=0}^{n-1}|X(k,l)|^2$ minus the specular component):

$$PSD = y(1-y)(n^4) \tag{2}$$

Since the aim with QR structures is to localise the power spectral density at the optical states within the target, then ideally the sum of the power spectral density at these optical states should be equal to PSD. Additionally, since we want the distribution of the power spectral density to be homogeneous, the power spectral density M at each optical state should then be:

$$M = \frac{y(1-y)(n^4)}{N} \tag{3}$$

where N is the total number of optical states within the target.

Equation 3 is equivalent to equation 4 in the main text. The optimisation target (equation 3 in main text) aims to minimise the total deviation between M and the real power spectral density at each optical state within the target. Finally, note that for a given array size n x n, according to equation 2 the non-specular power spectral density PSD is maximal at a fill factor $y = 0.5$.

Supplementary Discussion 4: QR unit cell designs

All the QR unit cell designs studied in this work are included in this section. All images include 9 unit cells in a 3 x 3 grid. White pixels correspond to entries equal to 1 in the unit cell array (which are assigned to SiO_2 once implemented in our device architecture), whereas black pixels correspond to 0s (Ag or $Al_{0.8}Ga_{0.2}As$ depending on whether a metallic or transparent design is considered). Note that the designs of QRE9-90 are equivalent to those of QRD9-90 with the same matrix sizes. However, since these QR families correspond to different spatial frequency targets, the pitch varies between the designs of QRE9-90 and those of QRD9-90. In QRE9-90, a pitch of 2.4 μm ensures that all OS between OS9 and OS90 are found within the target (from $k_{0,\ 800\ \mathrm{nm}}$ to $k_{\mathrm{GaAs},\ 900\ \mathrm{nm}}$). For QRD9-90, the target ranges from $k_{0,\ 600\ \mathrm{nm}}$ to $k_{\mathrm{GaAs},\ 700\ \mathrm{nm}}$, and it is at a pitch of 1.8 μm that all OS between OS9 and OS90 are found within these limits.

Finally, it is also important to mention that the designs for QRB5-26 and QRC5-37 with fill factors 0.4 are equivalent to those of the same QR families with fill factor 0.6, but with swapped entries in the unit cell array (black areas are swapped for white and vice versa). These 'reciprocal' structures have equivalent non-specular power spectral densities.

QRA1-2

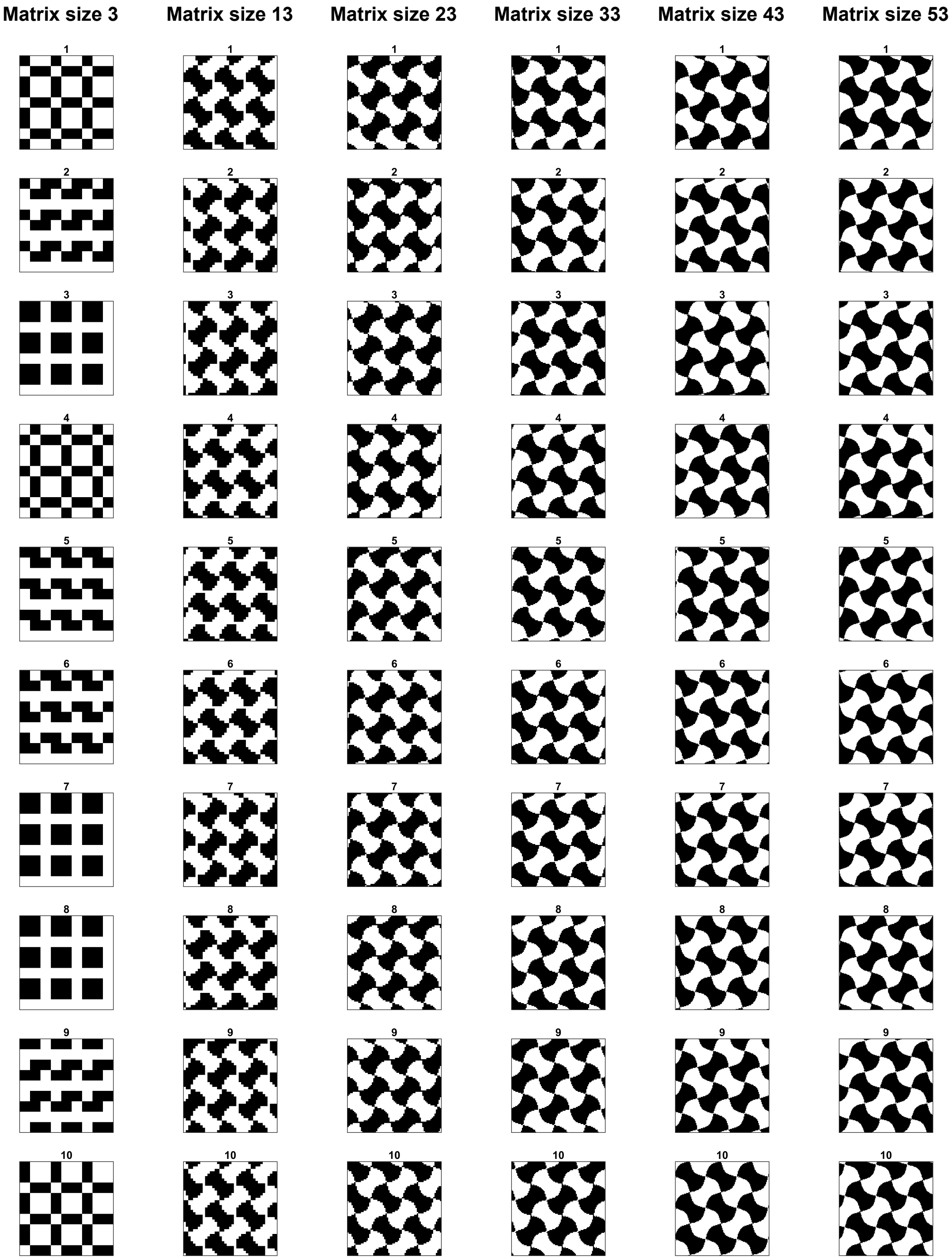
Matrix size 3
Matrix size 13
Matrix size 23
Matrix size 33
Matrix size 43
Matrix size 53

QRA5-16

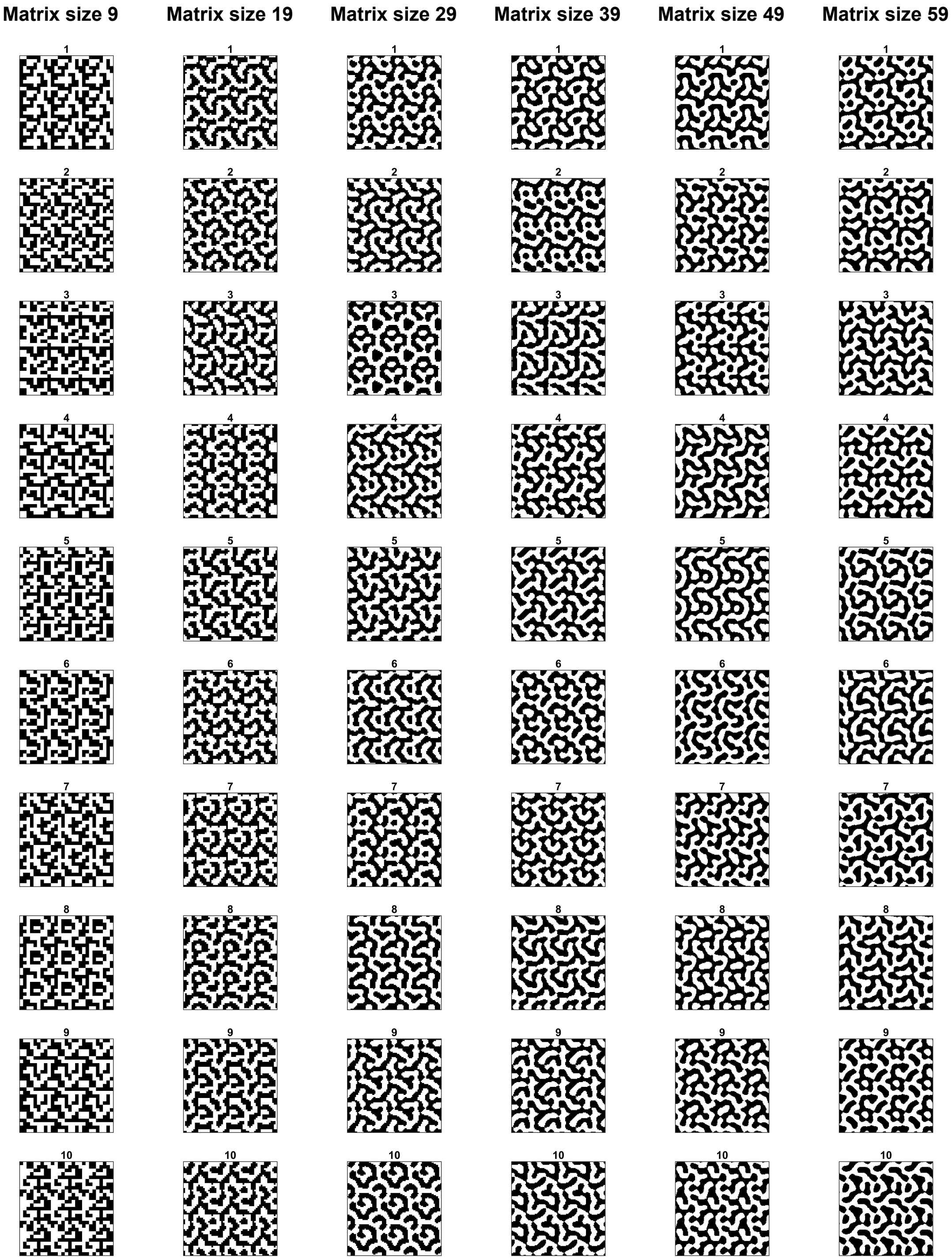
Matrix size 9
Matrix size 19
Matrix size 29
Matrix size 39
Matrix size 49
Matrix size 59
1
2
3
4
5
6
7
8
9
10

QRA9-26

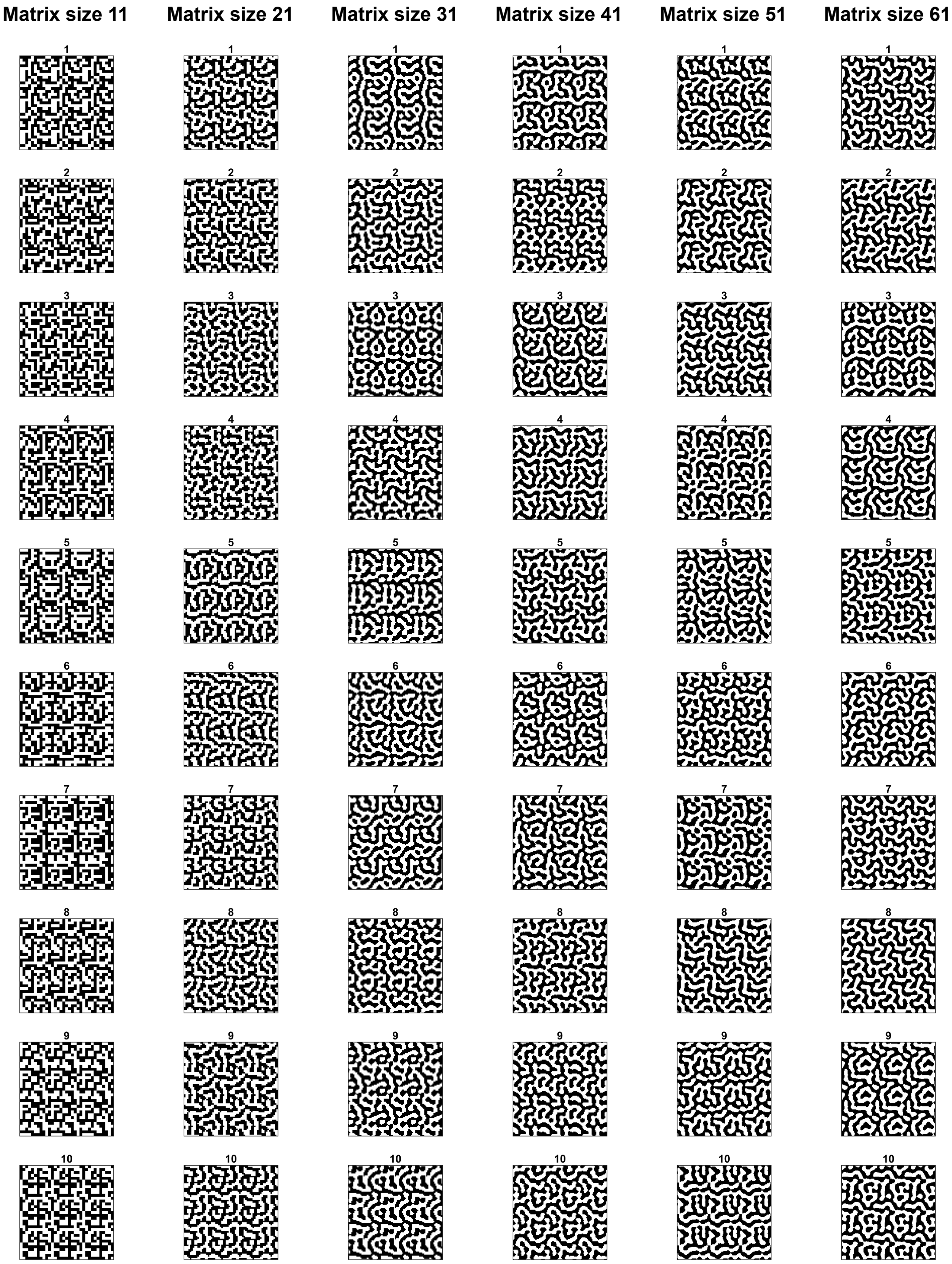
Matrix size 11
Matrix size 21
Matrix size 31
Matrix size 41
Matrix size 51
Matrix size 61
1
2
3
4
5
6
7
8
9
10

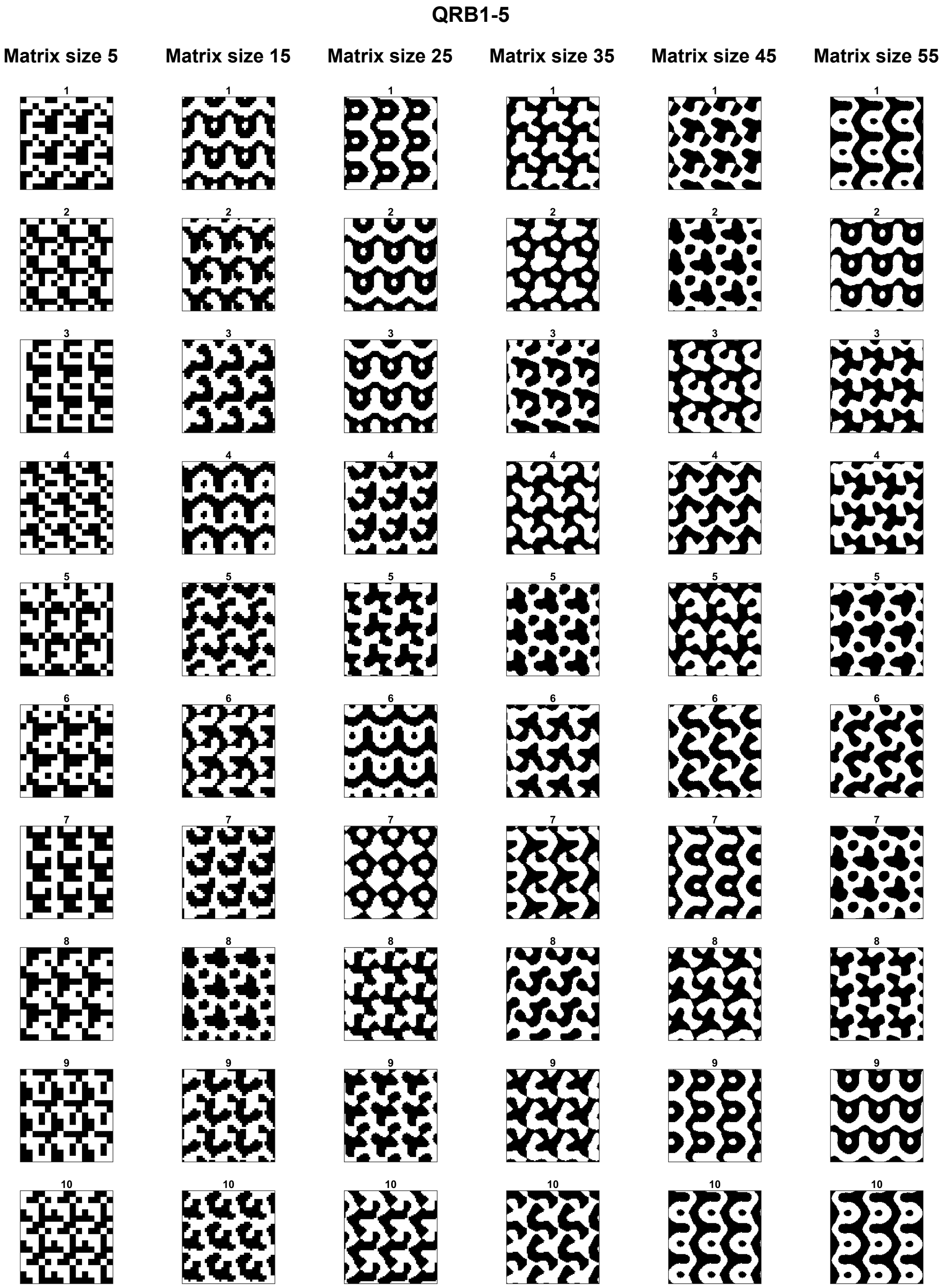
QRB1-5
Matrix size 5
Matrix size 15
Matrix size 25
Matrix size 35
Matrix size 45
Matrix size 55
1
2
3
4
5
6
7
8
9
10

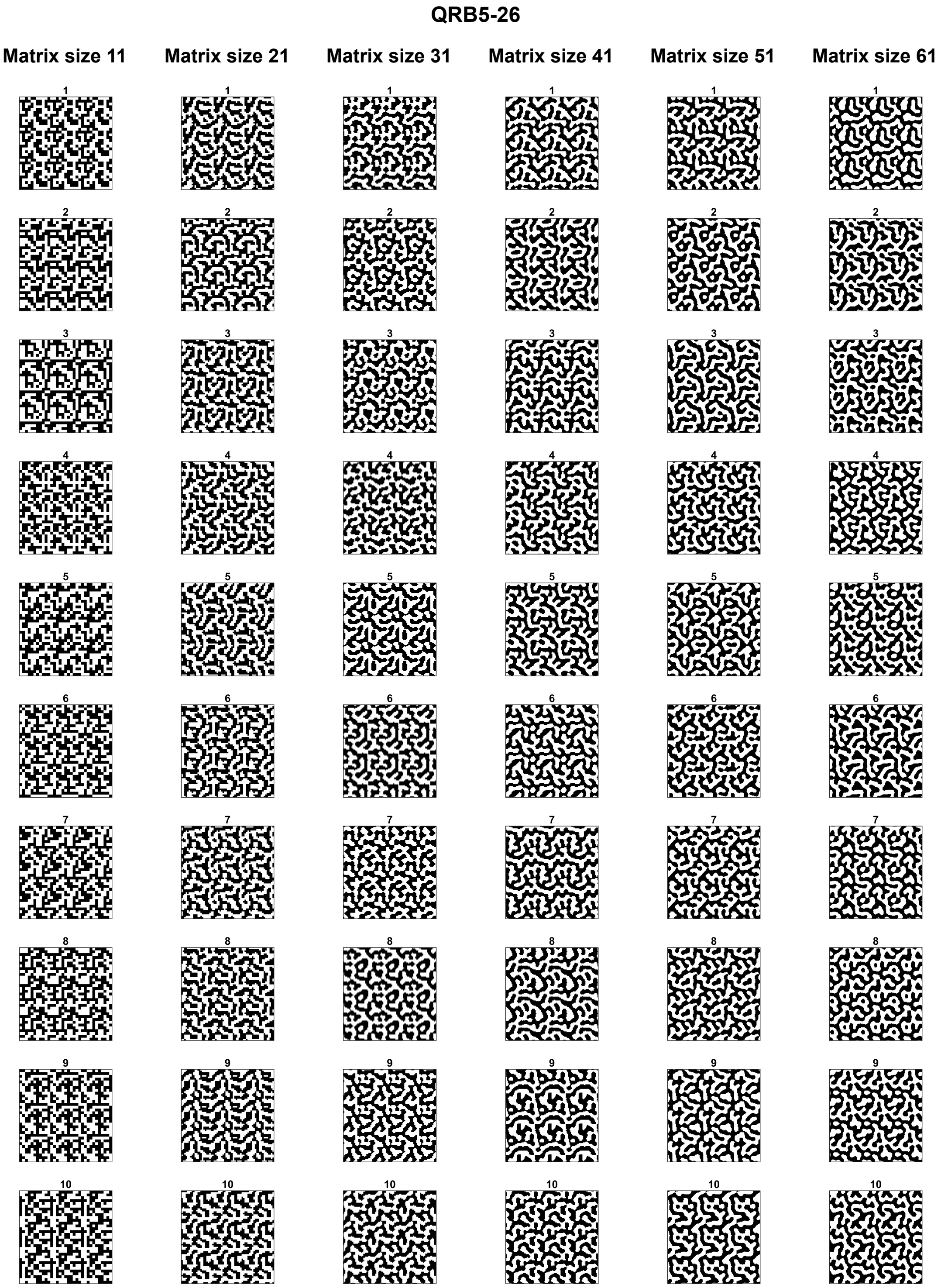
QRB5-26
Matrix size 11
Matrix size 21
Matrix size 31
Matrix size 41
Matrix size 51
Matrix size 61
1
2
3
4
5
6
7
8
9
10

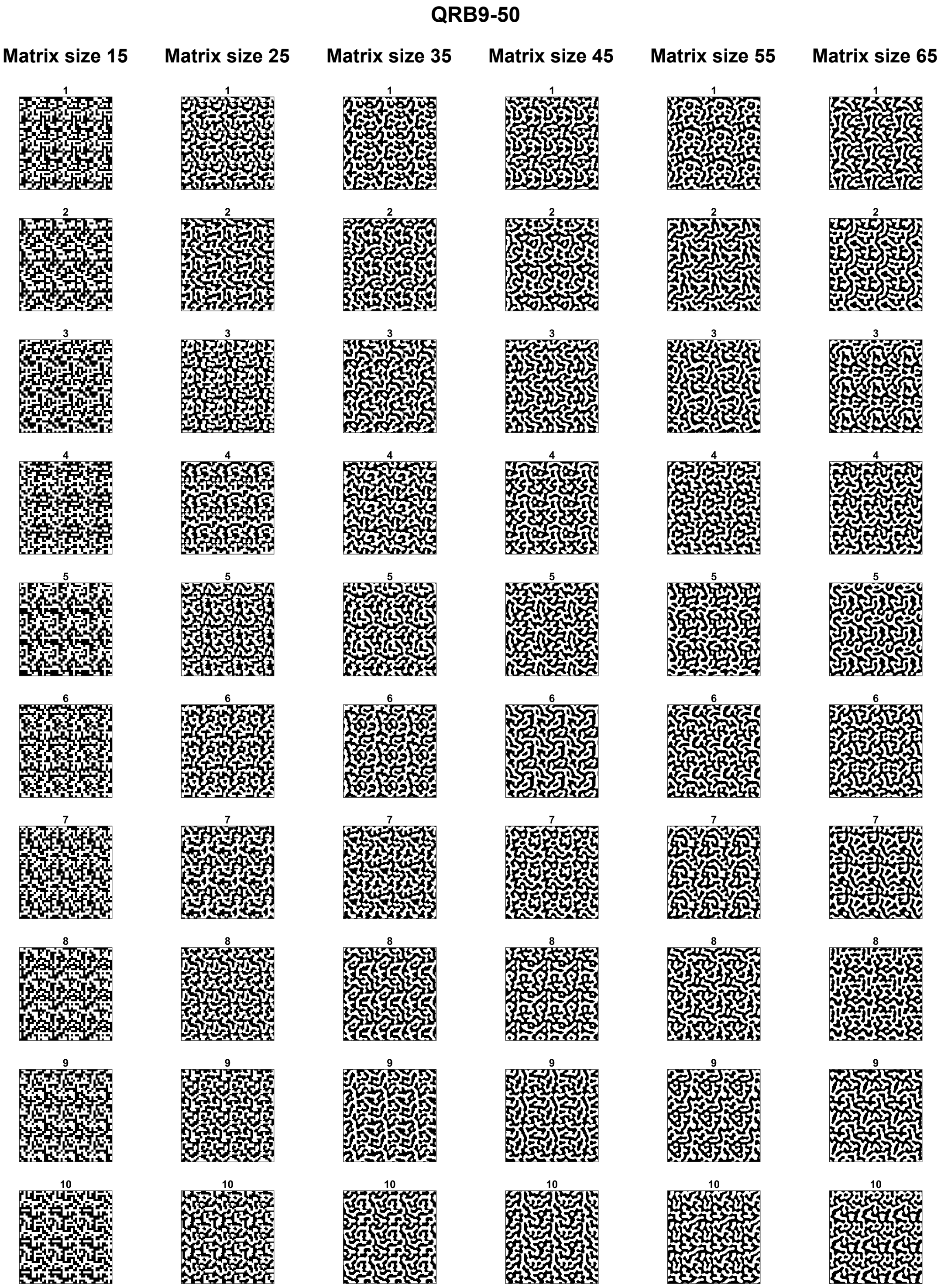
QRB9-50
Matrix size 15
Matrix size 25
Matrix size 35
Matrix size 45
Matrix size 55
Matrix size 65
1
2
3
4
5
6
7
8
9
10

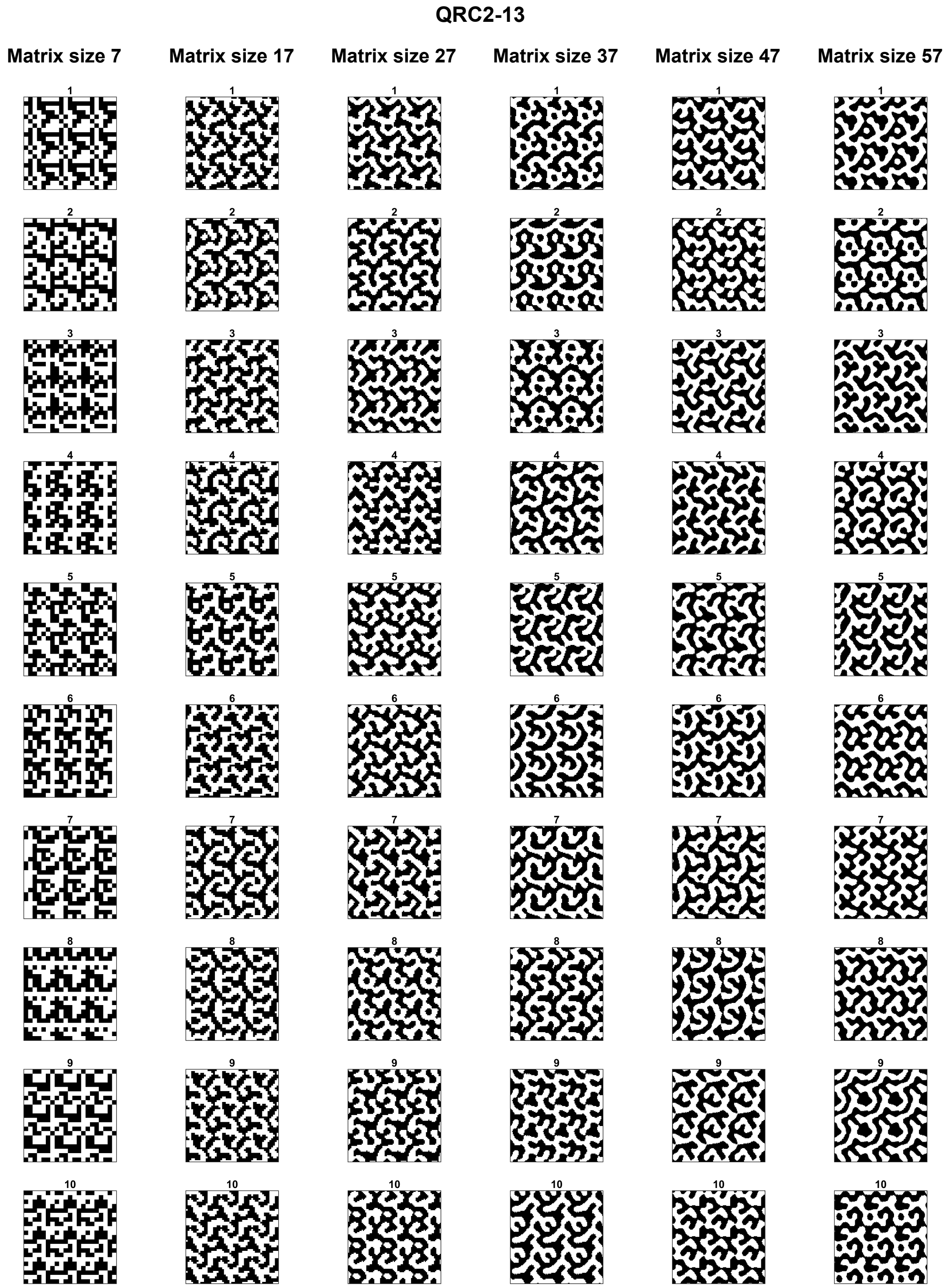
QRC2-13
Matrix size 7
Matrix size 17
Matrix size 27
Matrix size 37
Matrix size 47
Matrix size 57
1
2
3
4
5
6
7
8
9
10

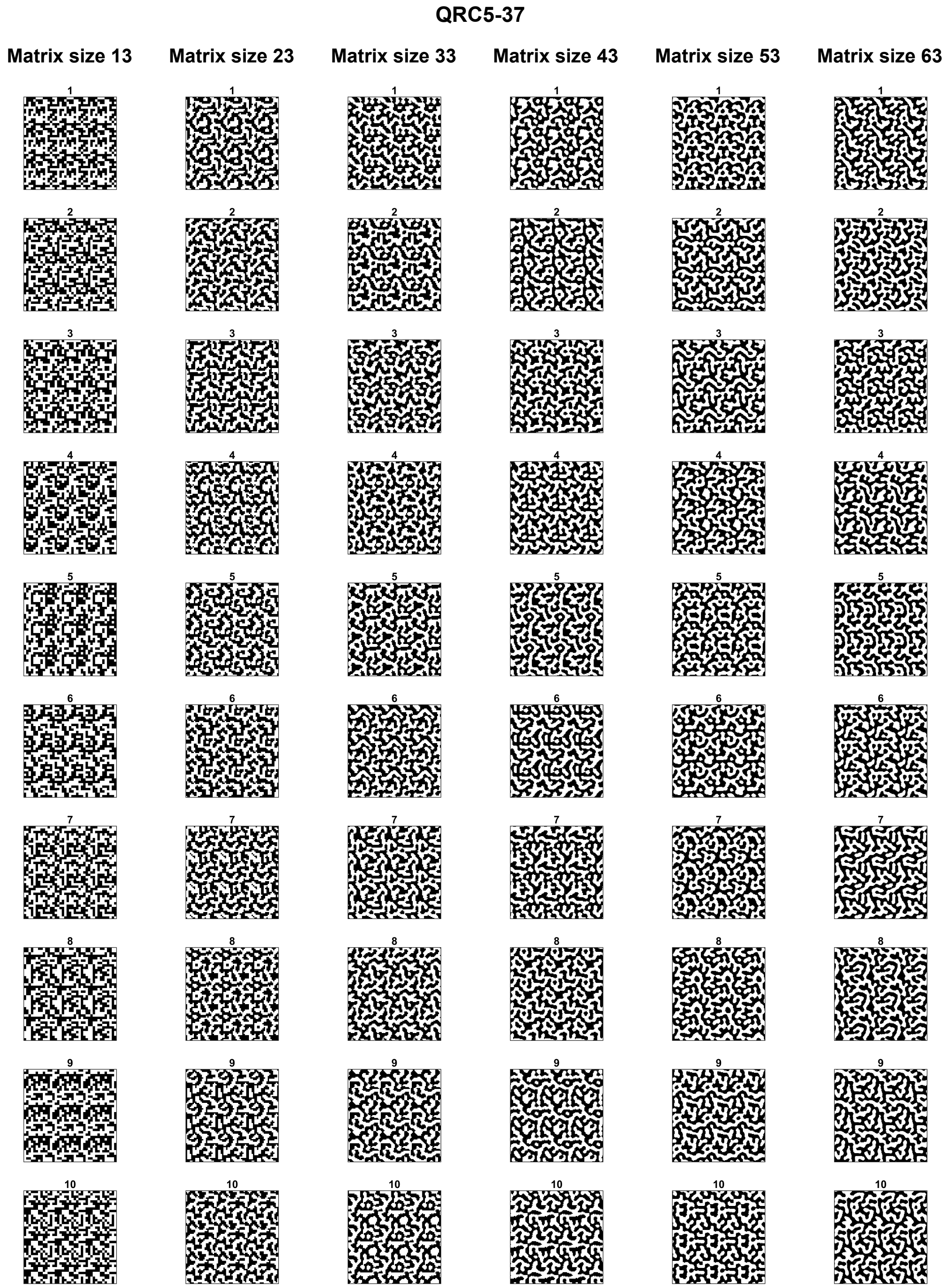
QRC5-37
Matrix size 13
Matrix size 23
Matrix size 33
Matrix size 43
Matrix size 53
Matrix size 63

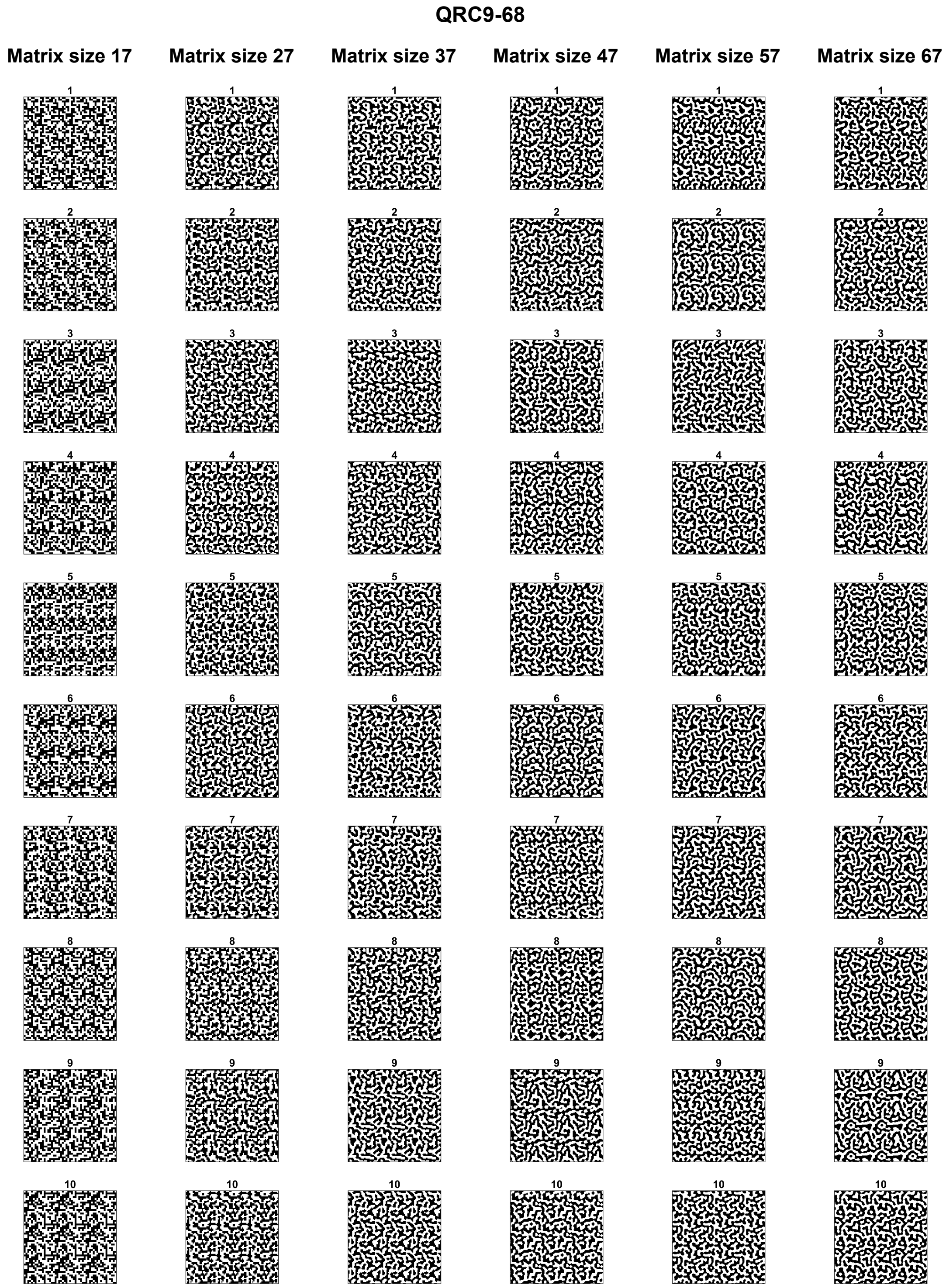
QRC9-68
Matrix size 17
Matrix size 27
Matrix size 37
Matrix size 47
Matrix size 57
Matrix size 67
1
2
3
4
5
6
7
8
9
10

QRD1-10

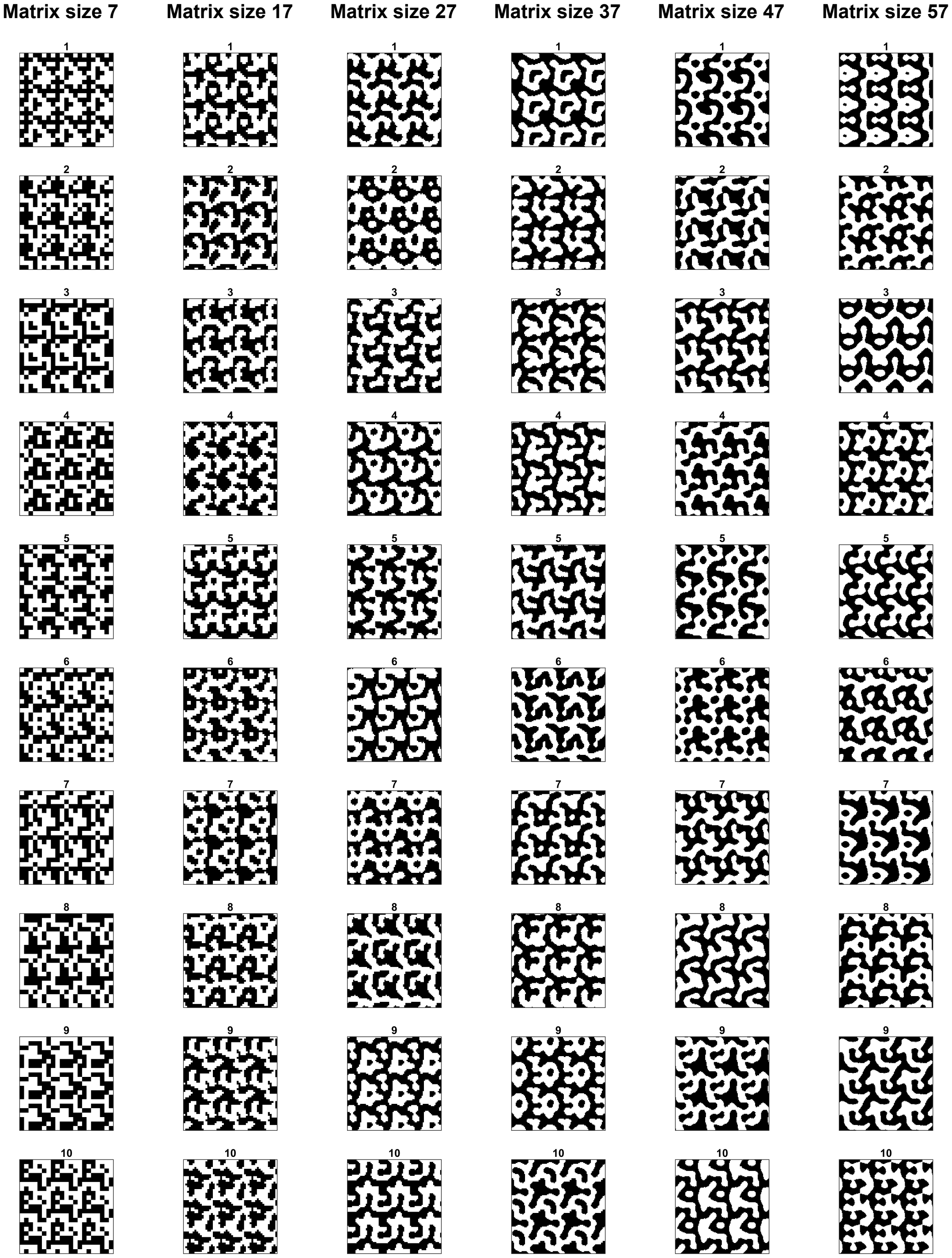
Matrix size 7
Matrix size 17
Matrix size 27
Matrix size 37
Matrix size 47
Matrix size 57

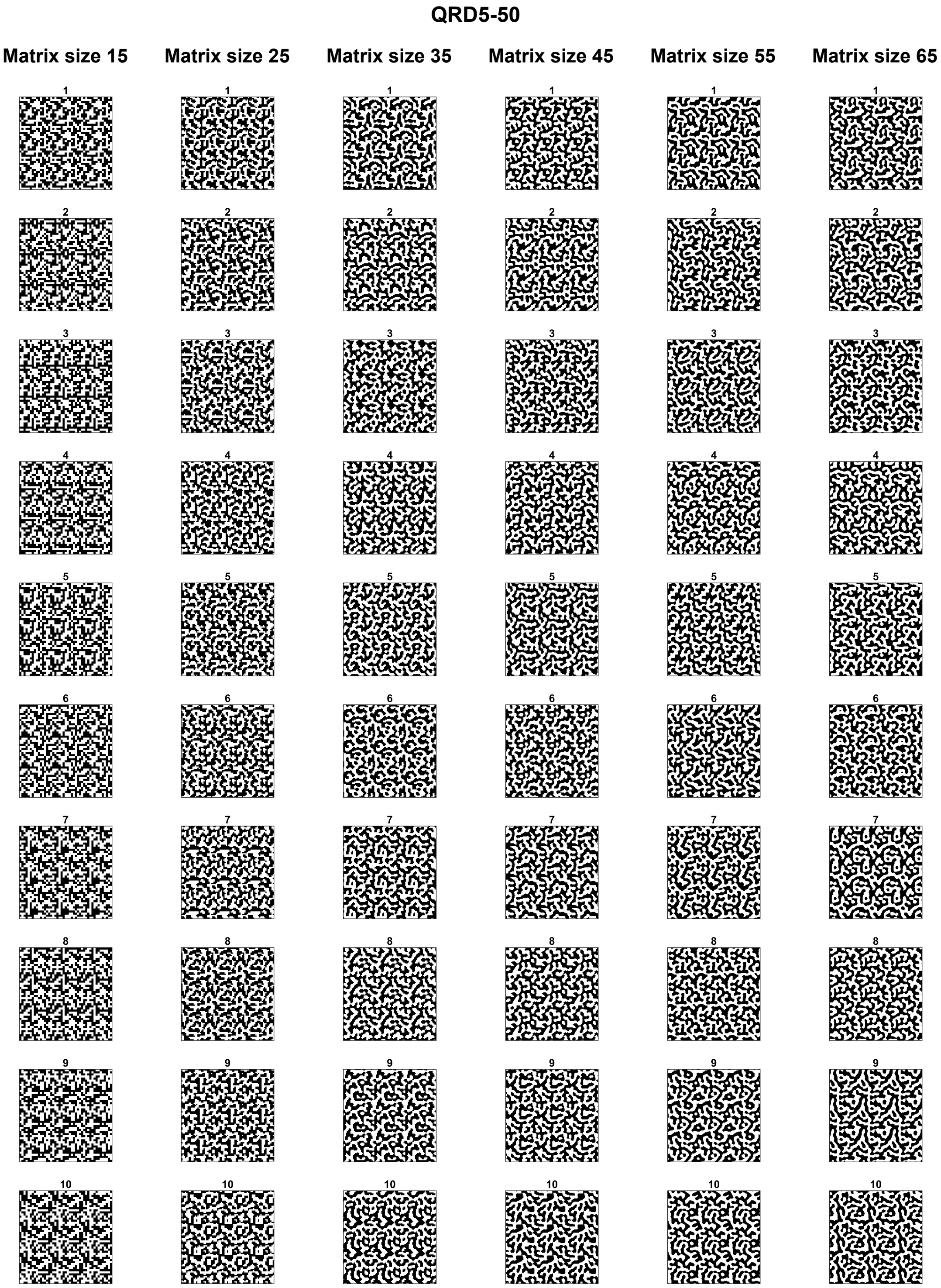
QRD5-50
Matrix size 15
Matrix size 25
Matrix size 35
Matrix size 45
Matrix size 55
Matrix size 65
1
2
3
4
5
6
7
8
9
10

QRD9-90

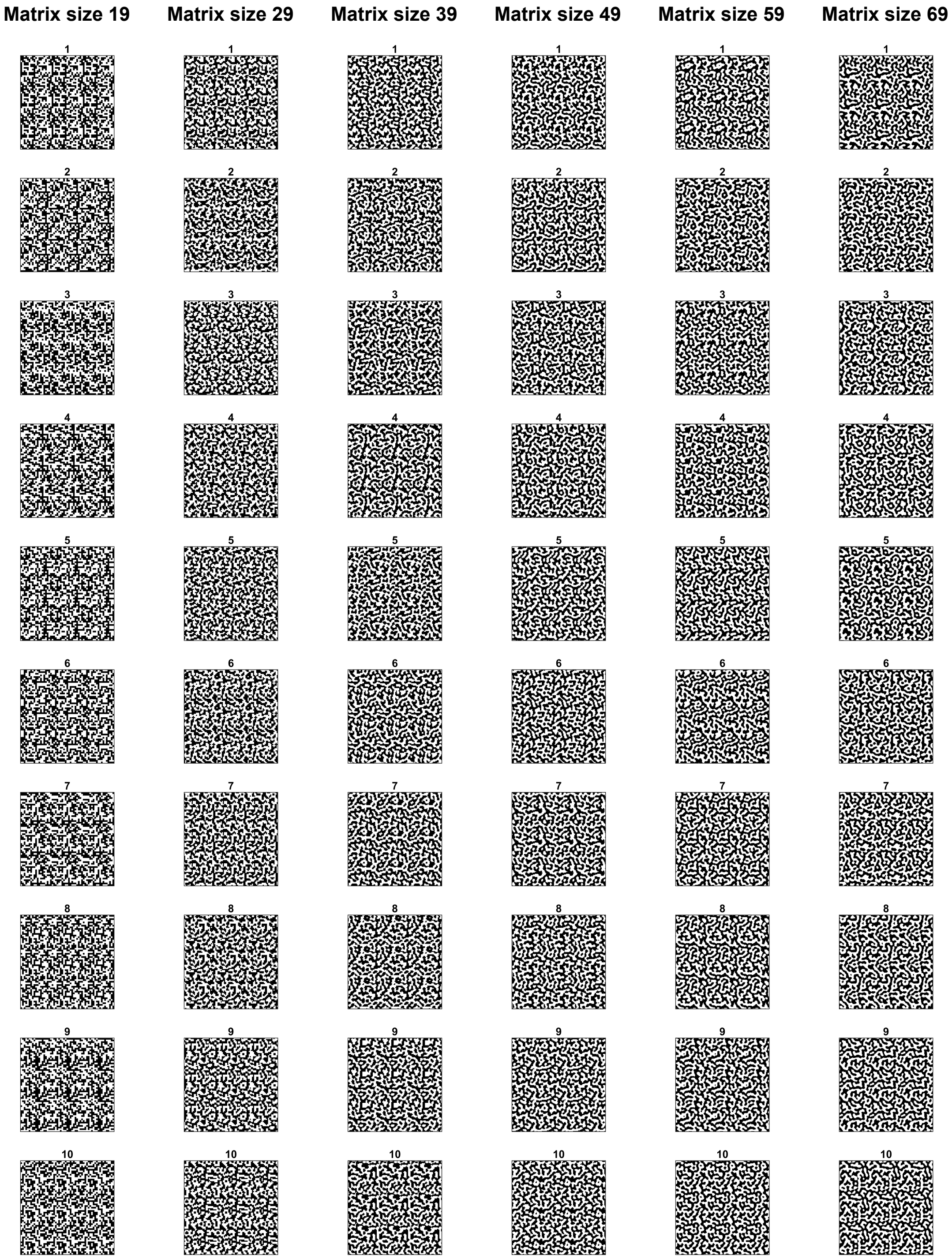
Matrix size 19
Matrix size 29
Matrix size 39
Matrix size 49
Matrix size 59
Matrix size 69
1
2
3
4
5
6
7
8
9
10

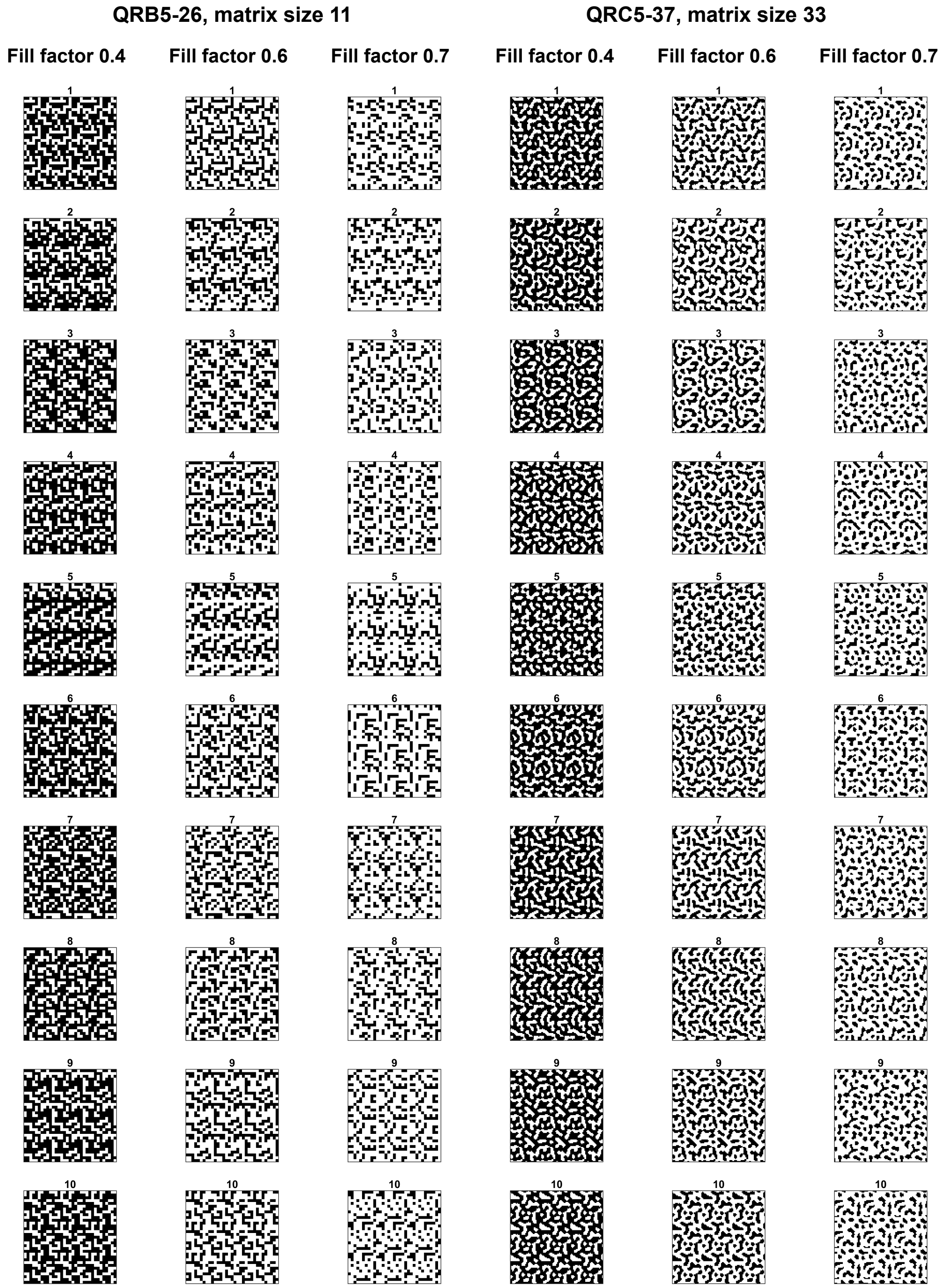
QRB5-26, matrix size 11
QRC5-37, matrix size 33
Fill factor 0.4
Fill factor 0.6
Fill factor 0.7
Fill factor 0.4
Fill factor 0.6
Fill factor 0.7
1
2
3
4
5
6
7
8
9
10

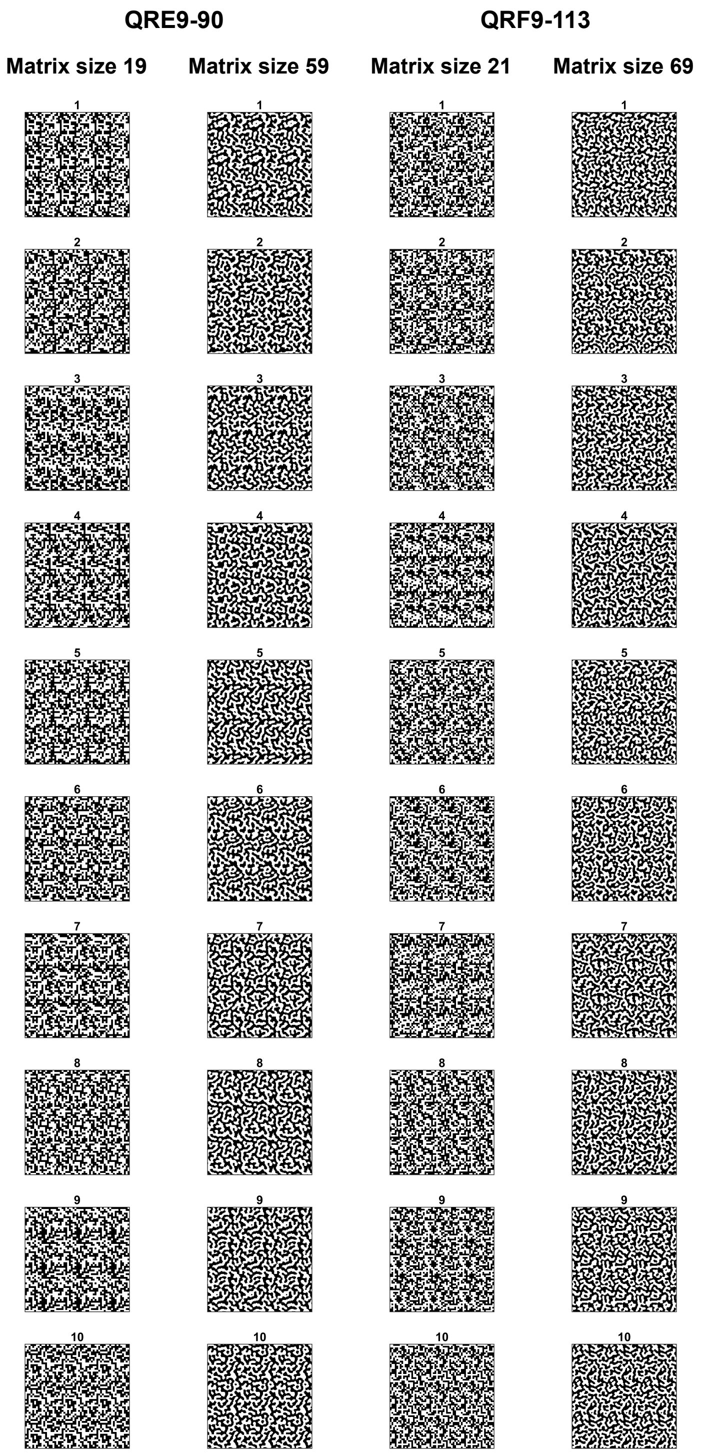
QRE9-90
QRF9-113
Matrix size 19
Matrix size 59
Matrix size 21
Matrix size 69

Supplementary Discussion 5: Fill factor studies in QR designs

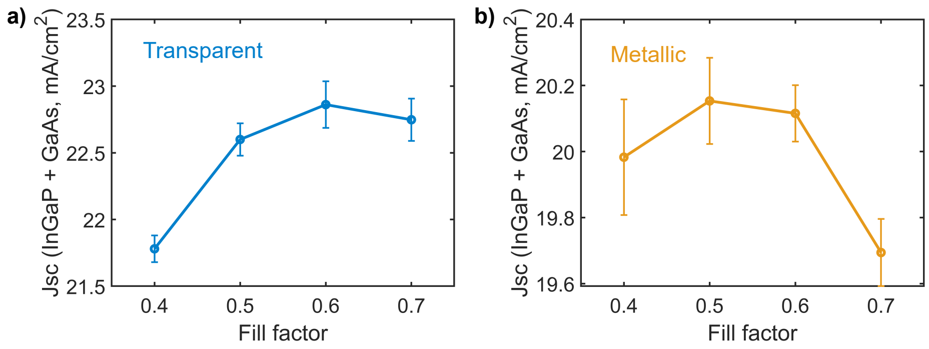

Figure S11: a) Average performance of ultra-thin devices with transparent QR gratings designed for QRC5-37 (matrix size 33 x 33) at different fill factors. For each fill factor, the average performance of 10 different unit cell designs is shown with error bars indicating the deviation within this population. b) Average performance of ultra-thin devices with metallic QR gratings designed for QRB5-26 (matrix size 11 x 11) at different fill factors. For each fill factor, the average performance of 10 different unit cell designs is shown with error bars indicating the deviation within this population.

QR unit cells were designed and evaluated at different fill factors for the best performing target, QR family and spatial resolution found for metallic and transparent device concepts in Fig. 3 and 4 (main text). The design algorithm was the one described in Methods (main text) with the appropriate fill factor. To evaluate the performance of these designs, for the transparent case the ARC thickness was fixed at 100 nm and grating thickness was optimised at each fill factor, being 100, 100, 120 and 140 nm for fill factor 0.4, 0.5, 0.6 and 0.7, respectively. For the metallic case, ARC thickness was fixed at 80 nm and grating thickness was also optimised at each fill factor, being 140 nm in all cases. The performance of 10 QR grating designs at different fill factors and with these optimal parameters is shown in Fig. S11.

Supplementary Discussion 6: Effect of increased spatial resolution on the parasitic losses in metallic QR textures

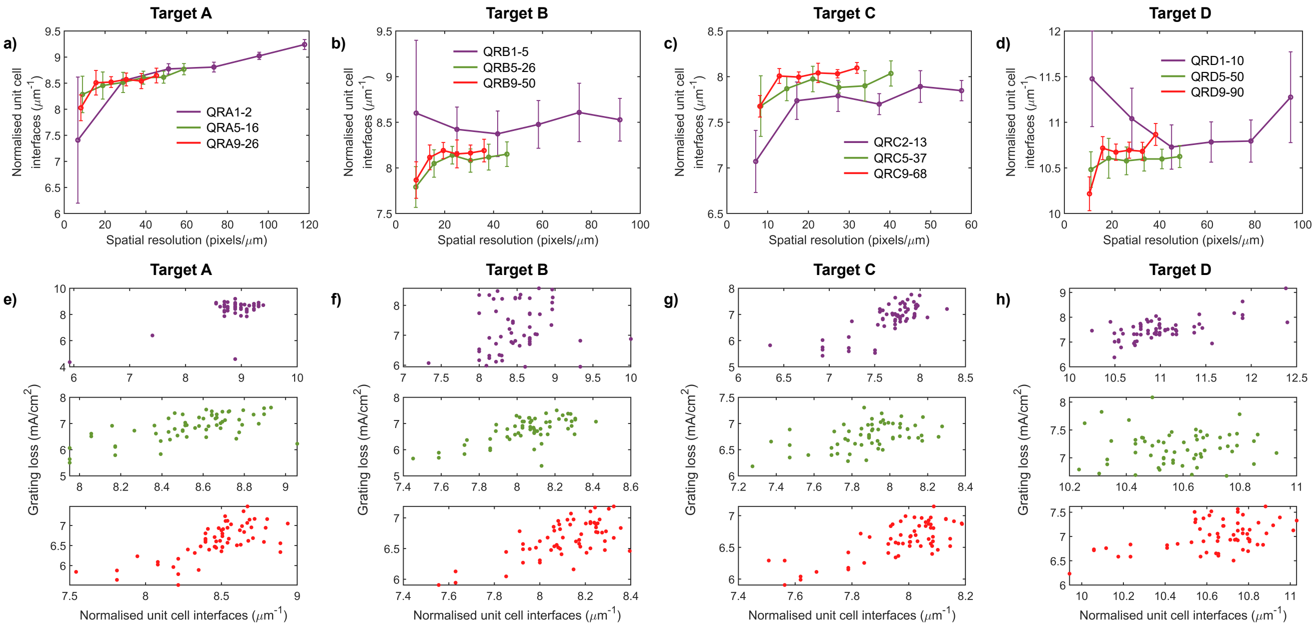

Figure S12: a-d) Average interfaces of all 10 unit cells designed for different spatial resolutions and QR families in Targets A, B, C and D at fill factor = 0.5. e-h) Correlation between the interfaces in each unit cell studied in a-d and the corresponding parasitic loss in the light trapping layer when such design is implemented with the metallic material selection.

It was observed in our study that metallic QR structures with increased spatial resolution generally led to lower device performance and higher losses in the light trapping layer. This observation may be due to different mechanisms. For example, inspection of the QR designs studied in our work (Supplementary Discussion 4) reveals that increasing the resolution of the unit cell leads to more intricate and detailed features, generally adding more interfaces between both grating materials. This is shown in Fig. S12 a-d, which include the average interface lengths between both texture materials in all 10 unit cells designed for different spatial resolutions and QR families in Targets A, B, C and D at fill factor 0.5. To obtain these data and for each QR design, the lengths of the interfaces between areas corresponding to different materials in one unit cell were calculated and added. The sum of all interface lengths was then normalised by the area of the corresponding unit cell design. For metallic QR gratings, the implications of the results in Fig. S12 a-d would be a general increment in the number of metallic edges and interfaces between the Ag and the SiO_2 as resolution is increased. These discontinuities enable field interactions with the metal where power can be lost parasitically. By becoming more abundant, such discontinuities could potentially drive an increase in the parasitic losses of the light trapping layer.

To further study this proposition, Fig. S12 e-h show the correlation between the 'normalised interfaces' of each QR design and the parasitic losses it experiences when implemented as the light trapping layer of our ultra-thin devices of interest. Note that these calculations were done considering the optimal parameters found in Table 2 (main text) and the metallic material combination (Ag/SiO_2). Losses are defined as the photocurrent that could

be generated by the parasitic absorption in the light trapping layer. Although no conclusive trends are observed, in some instances some correlation is observed between an increased number of interfaces and higher parasitic losses.

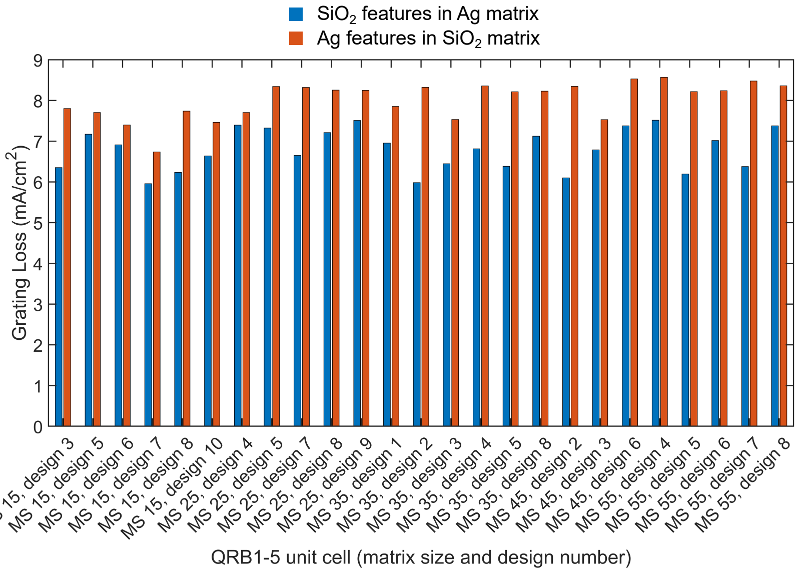

Figure S13: Parasitic losses in certain QR gratings designed for QRB1-5 when implemented with Ag and SiO_2. Designs are labelled according to Supplementary Discussion 4 and are those that contain distinct, isolated features in the unit cell. Parasitic losses are calculated as the photocurrent that could be generated with all the photons that are absorbed in the light trapping layer, considering devices with the optimal parameters in Table 2 (main text). Two scenarios are shown: having dielectric features in a metallic medium and vice versa.

Another mechanism that has a significant impact on the parasitic losses in the metallic light trapping layer is the real space arrangement of the materials in the grating. An example is found in the designs of QRB1-5, where high performance deviations are observed among unit cells for the same spatial resolution (Fig. 4 b in main text). The unit cells for QRB1-5 have distinct features as opposed to the intricate and interconnected channels in most other QR families (see Supplementary Discussion 4). The performance variations of these unit cells are linked to whether the features are made of Ag or SiO_2. Having dielectric features within a metallic matrix shows significantly reduced losses in the grating compared to the opposite case.

To highlight this observation, we ran simulations of some of these QRB1-5 designs for two possible cases: having dielectric features within a metallic matrix and vice versa (swapping 1s and 0s in the unit cell array to obtain inverse structures). Our results are shown in Fig. S13. In all cases, the same design (with the same amount of edges and interfaces) leads to lower parasitic losses in the light trapping layer if the features are dielectric. Loss variations between inverse designs can reach up to 2 mA/cm^2. Note that swapping the materials (i.e. swapping 1s and 0s) does not significantly affect the fill factor of the QR design (which is 0.5, or very close to this value considering the limitations imposed by the unit cell array size), nor the power spectral density characteristics. Although identified for QRB1-5, this loss mechanism is also expected to play a role in metallic gratings for other QR families. However, the increased intricacy of most designs as resolution is increased impedes performing a clear analysis of the relationship between real space geometry and power losses.

Supplementary Discussion 7: Estimation of achievable photovoltaic efficiency with optimal transparent QR structures

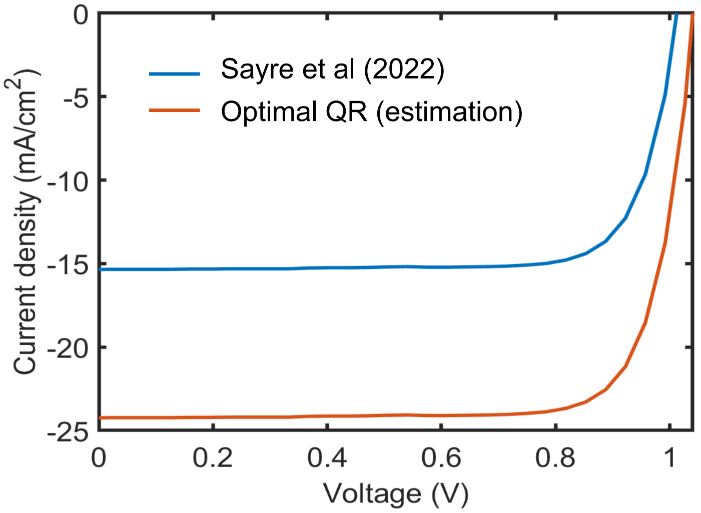

Figure S14: Experimental light IV curve of the hero nanophotonic device in [3] (blue curve), and estimated light IV curve for the devices in our current study with an optimal QR structure and double-layer antireflection coating (red curve).

In our 80 nm GaAs solar cells of interest and with an optimal double layer antireflection coating, one of our optimal transparent QR designs offers a simulated Jsc of 24.24 mA/cm^2. To estimate the photovoltaic efficiency that could be achieved with such Jsc, we take the light IV curve of the hero device obtained in [3] (blue line in Fig. S14) and shift it to match the Jsc of our optimal QR design (red line in Fig. S14). The Voc (1.04 V), FF (79.40%) and Jsc (24.24 mA/cm^2) of this shifted curve are then used to estimate the achievable photovoltaic efficiency of 20% with QR textures (for the AM1.5 solar spectrum). This approach is reasonable considering that the device architecture in [3] is equivalent to the one in our study, so we expect these cells to have comparable electrical performances. Shifting the light IV to the simulated Jsc is also reasonable from the perspective of the superposition principle.

Supplementary Discussion 8: State-of-the-art performances for thin and ultra-thin GaAs solar cells

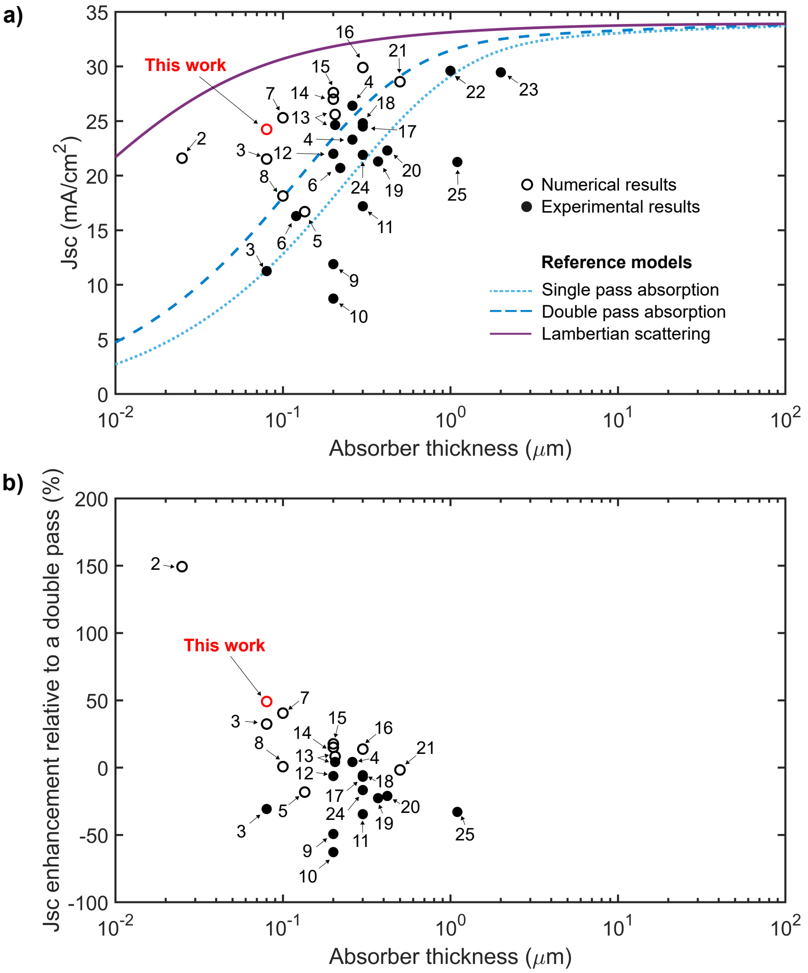

Figure S15: State-of-the-art performances of thin and ultra-thin GaAs solar cells (adapted from [1]). a) Jsc achieved by numerical and experimental studies on devices with varied absorber thicknesses. b) Jsc enhancement of the textured devices in a compared to the double pass reference model. Data points are numbered according to the reference list of this document.

To put the findings of our work in the context of the available literature, Fig. S15 a (adapted from [1]) shows a comprehensive snapshot of state-of-the-art performances (Jsc) for thin and ultra-thin GaAs devices. The figure shows results for both textured and planar devices, and highlights the performance of one of the optimal QR designs identified in our study. Although Fig. S15 a shows the competitiveness of our light trapping strategy, we include Fig. S15 b to better appreciate its absorption enhancement potential. This new figure shows the Jsc enhancement

achieved by the textured devices in Fig. S15 a, relative to the Jsc that is achievable with the double pass reference model. The enhancement offered by the optimal QR texture found in our work is one of the highest reported to date, obtained within a realistic device architecture and with a structure that holds unique engineering tolerance.

Supplementary Discussion 9: Convergence of RCWA simulations of QR structures

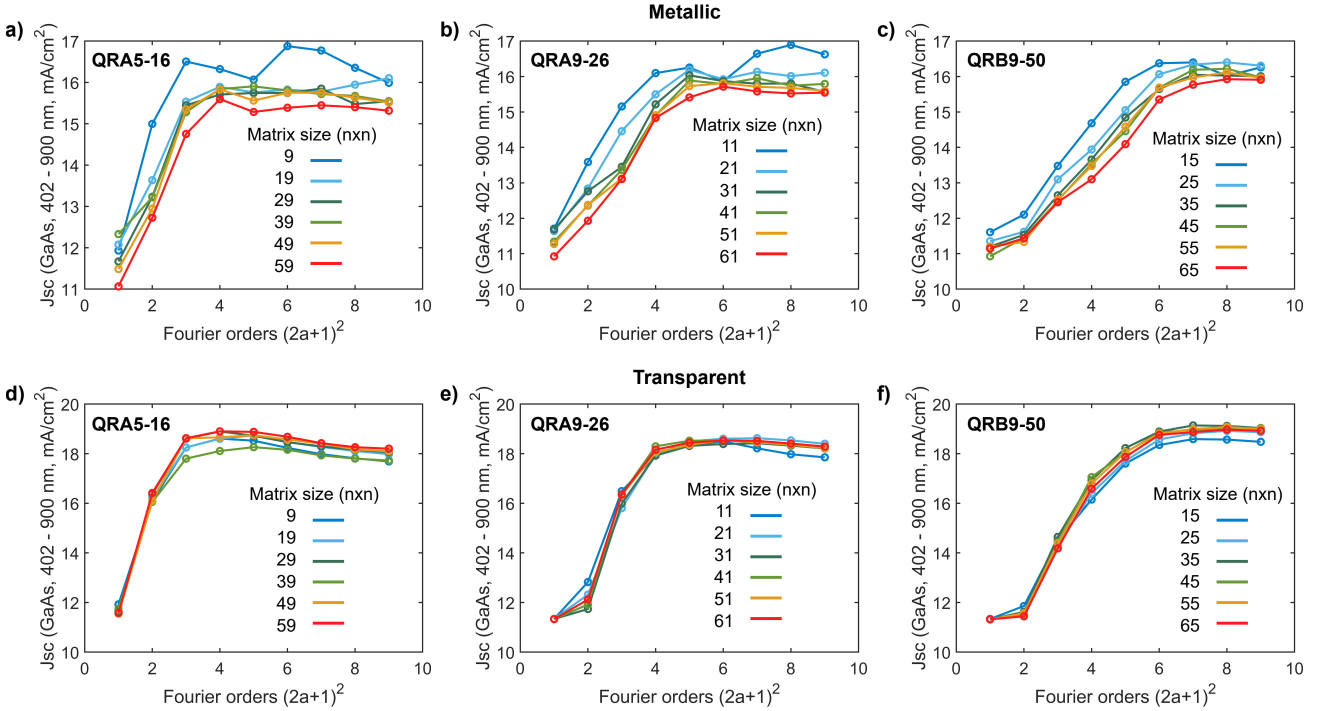

Figure S16: Jsc (calculated just considering the GaAs absorption from λ = 402 to 900 nm) as a function of the number of Fourier orders considered in the RCWA simulations, for unit cells of different QR families and array sizes (i.e. spatial resolutions) in a-c) metallic and d-f) transparent designs. Jsc is calculated considering the optimal parameters in Table 2 (main text).

The number of Fourier orders covered by the rectangular truncation scheme used in the RCWA simulations is equal to $(2a + 1)^2$, so that the optical states considered by the truncation span from $-a$ to a in both m_1 and m_2 (see equation 1 in main text). With both metallic and transparent QR structures, it was observed that absorption simulations could reach reasonable convergence as long as the range defined by a was large enough to encompass all the OS within the target spatial frequency region.

As an example, a unit cell for QRA5-16 has all optical states from OS5 to OS16 within the target. The minimum value of a that can cover all these optical states is 4. Fig. S16 a and d show the Jsc calculated with different numbers of Fourier orders for representative unit cells of QRA5-16, at different matrix sizes and for metallic and transparent structures. Note that good convergence is reached at the point where $a = 4$ in both cases. More examples are shown in Fig. S16 b, c, e and f for QRA9-26 and QRB9-50. In these cases, minimum values a of 5 (QRA9-26) and 7 (QRB9-50) are needed to cover all optical states within the corresponding targets. These are also the a values at which the Jsc of the representative devices reaches good convergence for different matrix sizes in both metallic and transparent cases.

These results show that simulation convergence is mainly dependent on the target under consideration in both

metallic and transparent QR designs. This target contains most of the nonspecular power spectral density and so it is expected that most of the diffraction profile and the corresponding absorption contributions will be found within this target. Matrix size (spatial resolution) does not seem to have an impact on convergence trends (note however that the lowest matrix sizes in the metallic case show weaker convergence). Also note how, in the simulations corresponding to the metallic scattering structures, the performance ranking is similar throughout the range of Fourier orders studied, with the highest spatial resolution (largest matrix size) consistently showing the worst performance.

In practice, 225 Fourier orders ($a = 7$) were considered for the simulations of all QR unit cells, except for those corresponding to QRC9-68 ($a = 8$), QRD9-90 ($a = 9$), QRE9-90 ($a = 9$) and QRF9-113 ($a = 10$). These values of a apply to both metallic and transparent simulations, and can provide convergence in all cases following the considerations discussed in this section.

References

[1] Inès Massiot, Andrea Cattoni, and Stéphane Collin. "Progress and prospects for ultrathin solar cells". In: *Nat. Energy* 5.12 (2020), pp. 959–972.

[2] Inès Massiot et al. "Metal nanogrid for broadband multiresonant light-harvesting in ultrathin GaAs layers". In: *Acs Photonics* 1.9 (2014), pp. 878–884.

[3] Larkin Sayre et al. "Ultra-thin GaAs solar cells with nanophotonic metal-dielectric diffraction gratings fabricated with displacement Talbot lithography". In: *Prog. Photovoltaics* (2021).

[4] Jeronimo Buencuerpo et al. "Efficient light-trapping in ultrathin GaAs solar cells using quasi-random photonic crystals". In: *Nano Energy* (2022), p. 107080.

[5] Jianling Xiao et al. "Paths to light trapping in thin film GaAs solar cells". In: *Opt. Express* 26.6 (2018), A341–A351.

[6] Nicolas Vandamme et al. "Ultrathin GaAs solar cells with a silver back mirror". In: *IEEE Journal of photovoltaics* 5.2 (2014), pp. 565–570.

[7] Jeronimo Buencuerpo et al. "Engineering the reciprocal space for ultrathin GaAs solar cells". In: *Opt. Laser Technol.* 142 (2021), p. 107224.

[8] Jonathan Grandidier et al. "Gallium arsenide solar cell absorption enhancement using whispering gallery modes of dielectric nanospheres". In: *IEEE Journal of Photovoltaics* 2.2 (2012), pp. 123–128.

[9] Keisuke Nakayama, Katsuaki Tanabe, and Harry A Atwater. "Plasmonic nanoparticle enhanced light absorption in GaAs solar cells". In: *Applied Physics Letters* 93.12 (2008), p. 121904.

[10] Wen Liu et al. "Surface plasmon enhanced GaAs thin film solar cells". In: *Solar Energy Materials and Solar Cells* 95.2 (2011), pp. 693–698.

[11] Weiquan Yang et al. "Ultra-thin GaAs single-junction solar cells integrated with lattice-matched ZnSe as a reflective back scattering layer". In: *2012 38th IEEE Photovoltaic Specialists Conference*. IEEE. 2012, pp. 000978–000981.

[12] Sung-Min Lee et al. "High performance ultrathin GaAs solar cells enabled with heterogeneously integrated dielectric periodic nanostructures". In: *ACS Nano* 9.10 (2015), pp. 10356–10365.

[13] Hung-Ling Chen et al. "A 19.9%-efficient ultrathin solar cell based on a 205-nm-thick GaAs absorber and a silver nanostructured back mirror". In: *Nat. Energy* 4.9 (2019), pp. 761–767.

[14] Sergey Eyderman, Alexei Deinega, and Sajeev John. "Near perfect solar absorption in ultra-thin-film GaAs photonic crystals". In: *Journal of Materials Chemistry A* 2.3 (2014), pp. 761–769.

[15] Sergey Eyderman and Sajeev John. "Light-trapping and recycling for extraordinary power conversion in ultra-thin gallium-arsenide solar cells". In: *Scientific reports* 6.1 (2016), pp. 1–7.

[16] Jeronimo Buencuerpo, Myles A Steiner, and Adele C Tamboli. "Optically-thick 300 nm GaAs solar cells using adjacent photonic crystals". In: *Opt. Express* 28.9 (2020), pp. 13845–13860.

[17] Weiquan Yang et al. "Ultra-thin GaAs single-junction solar cells integrated with a reflective back scattering layer". In: *Journal of Applied Physics* 115.20 (2014), p. 203105.

[18] Maarten van Eerden et al. "A facile light-trapping approach for ultrathin GaAs solar cells using wet chemical etching". In: *Prog. Photovoltaics* 28.3 (2020), pp. 200–209.

[19] Ingvar Åberg et al. "A GaAs nanowire array solar cell with 15.3% efficiency at 1 sun". In: *IEEE Journal of photovoltaics* 6.1 (2015), pp. 185–190.

[20] Boju Gai et al. "Multilayer-grown ultrathin nanostructured GaAs solar cells as a cost-competitive materials platform for III–V photovoltaics". In: *ACS nano* 11.1 (2017), pp. 992–999.

[21] Hitoshi Sai et al. "Light absorption enhancement in thin-film GaAs solar cells with flattened light scattering substrates". In: *Journal of Applied Physics* 122.12 (2017), p. 123103.

[22] Brendan M Kayes et al. "27.6% conversion efficiency, a new record for single-junction solar cells under 1 sun illumination". In: *2011 37th IEEE Photovoltaic Specialists Conference*. IEEE. 2011, pp. 000004–000008.

[23] MA Steiner et al. "Optical enhancement of the open-circuit voltage in high quality GaAs solar cells". In: *Journal of Applied Physics* 113.12 (2013), p. 123109.

[24] Daan van der Woude et al. "Ultrathin GaAs solar cells with a high surface roughness GaP layer for light-trapping application". In: *Progress in Photovoltaics: Research and Applications* (2022).

[25] Julia R D'Rozario et al. "Thin Gallium Arsenide Solar Cells With Maskless Back Surface Reflectors". In: *IEEE Journal of Photovoltaics* 10.6 (2020), pp. 1681–1688.
